# A cross-sectional study examining perceptions of discriminatory behaviors experienced and witnessed by veterinary students undertaking clinical extra-mural studies

**DOI:** 10.3389/fvets.2023.940836

**Published:** 2023-04-28

**Authors:** Olivia S. Summers, Rebecca Medcalf, Katherine A. Hubbard, Charlotte S. McCarroll

**Affiliations:** ^1^Department of Comparative Biomedical Sciences, Faculty of Health and Medical Sciences, School of Veterinary Medicine, University of Surrey, Guildford, United Kingdom; ^2^Department of Sociology, Faculty of Arts and Social Sciences, University of Surrey, Guildford, United Kingdom

**Keywords:** discrimination, veterinary, ageism and age-based discrimination, ableism, racism, sexism, homophobia

## Abstract

**Introduction:**

Recent research showed that 29% of respondents in a survey of veterinary professionals reported experiencing self-described discrimination in their workplaces. Senior colleagues and clients were responsible for discriminatory behaviors. As part of their training, veterinary students are expected to undertake extra-mural study (EMS) within these same workplaces and are likely to be vulnerable to discrimination from senior colleagues and clients. This study's objectives were to identify and characterize the pattern of perceived discriminatory behaviors (i.e., belief of being treated unfairly) that veterinary students encounter while seeing practice and explore students' attitudes toward discrimination.

**Methods:**

Students at British and Irish veterinary schools who undertook some clinical EMS completed a survey of closed and open questions as part of a cross-sectional study. Demographic data and experiences of discrimination with details of incidents and reporting were collected, alongside respondent attitudes. Quantitative data were analyzed using Pearson's chi-squared analysis to analyse respondents' characteristics and their experiences of discriminatory behaviors and subsequent reporting. Qualitative content analysis was used for open-question data.

**Results:**

Of the 403 respondents, 36.0% had perceived behavior they believed was discriminatory. The most frequent form of discrimination was based on gender (38.0%), followed by ethnicity (15.7%). There were significant associations between respondents' experience of discriminatory behaviors and the following characteristics: age (*p* = 0.0096), disability (*p* < 0.00001), race/ethnicity (*p* < 0.0001), gender/sex (*p* = 0.018), and LGBTQ+ status (*p* = 0.001). Supervising veterinarians were the most commonly reported perpetrators of discriminatory behaviors (39.3%) compared with clients (36.4%). Only 13.9% of respondents who experienced discrimination reported the event(s). Respondents with a disability were the least likely to agree with the statement that professional bodies are doing enough to tackle discrimination (*p* < 0.0001). Most respondents agreed that sexism is still an issue (74.4%), but men were more likely to disagree (*p* = 0.004). Most respondents felt that ethnic diversity needed to be increased (96.3%).

**Discussion:**

Discriminatory behavior is a problem for students seeing practice, especially those with one or more protected characteristics (as defined by the UK Equality Act 2010). Improved education would need to include perspectives from minority groups to help remove discriminatory behavior from veterinary practice.

## 1. Introduction

There has been a multitude of studies examining different types of discrimination within the medical profession. The types of discrimination reported and studied were gender-based (1–5), race-based (6–9), and disability-based (10). When specifically examining medical education, discriminatory behavior typically consists of humiliating, hostile, or abusive behaviors by senior colleagues most commonly in the consultant role (11). Medical students who disproportionately experienced these discriminatory behaviors were female (12), lesbian, gay, bisexual, transgender, and queer plus (LGBTQ+) people (13), non-white (14), or any combination of these intersecting identities (15, 16). While gender and race are common themes for research into discrimination, there are fewer studies examining any other protected characteristics as defined by the UK Equality Act 2010 (17) namely age, religious beliefs, sexual orientation, marriage/civil partnership status, pregnancy/parental leave status, gender reassignment, and disability (17), or characteristics that are not specifically protected by the Act such as socioeconomic status.

Within the veterinary profession, there is much less information on discrimination available compared with other professions. The latest research within the context of the veterinary profession has identified gender-based discrimination despite an increasing number of female veterinary students (18). However, this research did not examine the full range of protected characteristics and did not take into account the intersecting marginalisations of participants. Recently, the British Veterinary Association (BVA) sought to address the paucity of information on discrimination in all areas under the UK Equality Act 2010. They aimed to address the said paucity with two studies; one surveying their membership and the other taking a cross-section of the veterinary profession including non-members, students, and other veterinary professionals (19). The combined report identified that nearly one-third (29%) of self-selecting respondents reported a total of 1,305 incidents of discriminatory behavior in a veterinary workplace (19). The main two discriminatory behaviors described in this report targeted gender at 43%, with race/ethnicity second at 26% (19) showing a similar pattern within the veterinary profession as that observed in the medical profession.

Veterinary medicine training in the UK requires students to undertake clinical and pre-clinical extra-mural studies (EMS) to further develop their practical skills and gain experience outside the course setting. EMS exposes students to potential discrimination from members of the public, other veterinary professionals, and other students. The BVA survey, which included responses from students making up 9% of the sample, found that the majority of discrimination incidents reported were perpetrated by a more senior colleague at 47% *vs*. 35% by clients (19). Since students are likely to spend more time with senior colleagues than with clients while on EMS, a student-based survey could yield similar results. Indeed, two recent studies conducted in the medical education environment found that it was senior colleagues and medical educators who were the primary perpetrators of discriminatory acts and behaviors (11, 20).

The BVA study highlighted a concerning statistic that only 19% of incidents experienced by students were reported (19). This could be due to a power imbalance between the student and placement provider which plays into the power aspect of the intergroup relation theoretical framework of discrimination (21). This framework describes how those in the majority or in-group have direct power over those in the minority or out-group. This power dynamic creates an environment such that the powerless are unlikely to speak up about discriminatory acts or behaviors. Students are likely to perceive themselves to be part of the powerless out-group in terms of being a new and temporary member of an established team, or as an inexperienced junior entering a team of experienced professionals. For students from marginalized groups, these intergroup relations are compounded. Furthermore, the BVA study also highlighted that less than half (45%) of the respondents were concerned about diversity in the profession (19). Some free-text expansions on this lack of concern were about semantics [e.g., on legal definitions of what the word “discrimination” means (19)]. These semantic arguments neglect the impact the behaviors not exceeding legal thresholds have on individuals. Whether a behavior is worthy of legal action is beside the point as the perception of the discriminatory behavior can still result in a negative psychological impact for an individual (22).

Intersecting marginalisations play a significant role in shaping the experiences of discrimination in medical students (16). Crenshaw discussed how the intersection of both race and gender can synergistically impact one another and compound the discrimination experienced by individuals with more than one marginalized identity, black women in the case of Crenshaw's work (23). By broadening the scope of this study to more than one characteristic of discrimination, it is hoped to highlight the experiences of veterinary students and provoke questions for further discussion and research.

This exploratory study has four aims. First, to determine the pattern of discriminatory behaviors that veterinary students experience and/or witness while seeing practice within the veterinary profession. Second, to explore the features of such discriminatory behaviors, including where they took place and who perpetrated the discriminatory behavior. Third, to identify what the common reasons for not reporting were and how that may relate to intergroup dynamics. Finally, to gain a cross-sectional snapshot of current student perceptions and attitudes regarding discrimination that can be used to identify future avenues of research and informing of policy. This study will examine age, disability status, race/ethnicity, gender, and LGBTQ+ identity as the main areas of focus applicable to students based on the student responses in the BVA study (19).

## 2. Materials and methods

### 2.1. Ethics

Full ethical approval was granted a favorable opinion by the University of Surrey Ethics Committee FHMS 19-20 023 EGA.

### 2.2. Study Design

This cross-sectional study adopted a similar design to the BVA Discrimination Survey of a series of closed and open survey-based questions focusing on the demographic characteristics respondents perceived to be targeted by perpetrators of negative behavior (19) but with a student-centric target population. While the UK medical student study by Broad et al. explored the type of behaviors, e.g., inappropriate jokes and favoring of individuals (20), the focus of this study was more on the characteristics of the participants, location, and perpetrator.

The target population was veterinary students in the UK and Ireland in their clinical years of study during the study period of 4th March−27th April 2020, and undertaking clinical EMS, i.e., students in their third year or greater gaining experience in a clinical veterinary workplace. Eligible participants were invited to take part in a cross-sectional study. The method used was an online survey using “Online surveys” software (Jisc, 2020) containing a combination of quantitative survey items, free-text responses, and Likert scales (Supplementary material). The target population was estimated to be 3,200, from the total number of veterinary students enrolled in British and Irish veterinary programmes at the time the study was conducted [5,295 (24)]. Students in their first or second year gaining experience in animal handling in a non-veterinary context (farm, stables, kennels, etc.) were not included in this study. It was calculated that a sample size of 320 would be required using the value of 29.5% experiencing or witnessing perceived discrimination based on the reported 721 respondents out of 2,445 (29.5%) in the BVA discrimination survey (19) and a confidence level of 95%. A survey link was emailed to eligible individuals at the University of Surrey *via* internal email and distributed to students at all other UK and Ireland veterinary schools *via* representatives of the Association of Veterinary Students. Participation was incentivised with the opportunity to enter a prize draw for retail vouchers. Exclusion criteria were students not within their clinical years of study and/or those reporting incidents of discriminatory behavior or comments not relating to clinical EMS as determined from the free-text description of incidents.

### 2.3. Data collection

Broad et al. concluded in their study of medical students at the University of Bristol that discrimination was associated specifically with gender, ethnicity, sexuality, disability, and year group so the current study's questions targeted these particular characteristics (20). Respondents were first questioned to determine the sample population demographics. Ethnic characteristics were based on Government census ethnicity categories (25). All other characteristics that participants may have perceived had been the cause of discriminatory behaviors, which were based on the list of “protected characteristics” included in the UK Equality Act 2010 (17).

To address the first aim of the study, which is to understand the discrimination experienced by veterinary students, respondents were asked whether they had personally experienced any behavior or comments they perceived to be discriminatory with simple yes, no, or not sure selections. A legal definition of “discrimination” was avoided in participant information material to get the respondents' perceptions of behavior experienced and/or witnessed as it is the perceptions that can have a negative psychological impact on an individual (22). Those who responded either “yes” or “not sure” were then asked to expand on the details of the incident(s) they experienced. Respondents were asked to select the category(ies) under which they believed incidents fell. Specifically, these were the protected characteristics as used in the UK Equality Act 2010: age, disability, gender identity/reassignment, marriage or civil partnership, pregnancy and maternity, race/ethnicity, religion/belief, sex/gender, sexual orientation, and other with an option to define a category outside of the Equality Act characteristics. Respondents were also asked about the frequency of discrimination incidents, profession sector(s) (e.g., companion animal, production animal, etc.), and role of the perpetrator(s) (e.g., veterinary surgeon, client, etc.), and whether they reported the incident(s) and their reasons for doing or not doing so. The content of free-text descriptions of incidents by respondents was assessed to determine if the respondent's interpretation of the question matched the inclusion/exclusion criteria, namely that incidents described are related to clinical EMS, and to confirm the category/categories of discrimination for analysis.

Common findings in the BVA Discrimination Survey and Broad et al.'s study identified that reporting of discriminatory behavior or comments was uncommon or rare (19, 20), so the current study aimed to ask a similar question and identify possible reasons why.

All respondents were asked to rate their agreement, using a Likert scale, regarding statements about discrimination against veterinary students to establish the general views of the student body.

Finally, students had the option to add any further comments regarding the survey subject in a free-text box. This was to allow the expression of views that the survey limited them in sharing and to gain further insight into their opinions on the topic.

### 2.4. Data analysis

Quantitative data were analyzed using IBM SPSS Statistics 27 for Windows (IBM Corporation) for descriptive statistics and Pearson's chi-squared test of association. The chi-squared test was chosen due to the data being categorical, with the Likert score selected being the dependent variable and the demographic group being the independent variable with an accepted significance level of 0.05. Confidence intervals for proportions were determined by the Binomial Exact Method (26) using a CI proportion calculator developed by UCSF Clinical & Translational Science Institute (27).

For analytic purposes, respondents were grouped by the demographic characteristics of age, disability status, ethnicity, gender, and sexual orientation. For age, respondents were categorized as under 24, 24–27, and over 27 years. Disability was grouped as those self-declaring either no disability or having a disability. Ethnicity was grouped into white British, white other (including Irish, European, and North American), and BAME (Black, Asian, and Minority Ethnic) to ensure sufficient *n* numbers for statistical analyses. Gender was grouped as female, male, and non-binary; however, only two respondents identified as non-binary, hence were insufficient for analysis. LGBTQ+ status was grouped on respondent answers to the sexual orientation and gender identity matching that were assigned at birth questions and so were grouped as heterosexual or LGBTQ+ (including lesbian, gay, bisexual, pansexual, transgender, queer, and any other stated sexuality or gender identity).

Specific types of discriminatory behavior or comments were grouped as ageism (targeted toward age), ableism (targeted toward disability status), racism/xenophobia (targeted to respondent's perceived ethnic origin or skin color), sexism (targeted toward gender, predominantly misogyny), homophobia (targeted toward sexual orientation), and LGBTQphobia (targeted toward any aspect of LGBTQ+ identity including homophobia, biphobia, and transphobia). Incidents described as affecting more than one characteristic were grouped within all the applicable categories. For example, if an incident referred to both a person's age and race, the incident was categorized in both the ageism and racism/xenophobia categories.

Likert scales were used where respondents could select one of five categories from strongly agree to strongly disagree with several statements presented (see Supplementary material). Confidence intervals and Pearson's chi-squared associations were tested for each statement and respondents' characteristics to determine if there was an association between respondents' level of agreement with statements and their demographic characteristics.

Responses to the open-ended questions describing incidents were qualitatively analyzed using content analysis. They were coded and grouped according to content (e.g., which protected characteristics were being referred to). These data both validated the quantitative data to confirm the correct category of type of discriminatory behavior or comments experienced or witnessed and provided frequencies for codes to draw out further insight. Selected quotes that illustrate frequent types of responses are provided and further examples can be found in Supplementary material.

## 3. Results

### 3.1. Demographics

A total of 407 individuals responded to the survey, this gives a response rate of 12.6% which is a relatively small sample size. Of those, four had described experiences of discrimination within their pre-clinical extra-mural studies so were excluded from the analysis leaving 403 responses. Most respondents were under 24 years of age, had no disability, were white British, were female, and identified as heterosexual (Table 1).

**Table 1:** Demographics of respondents.

**Demographic**	**Number of respondents**
**Age**	
Under 24	289
24–27	95
Over 24	19
**Disability status**	
No disability	364
Disability	35
Prefer not to say	4
**Race/Ethnicity**	
White British	294
White Other	61
White Asian	8
Chinese	16
Indian	6
African or Caribbean	6
Mixed/Other	11
Prefer not to say	1
**Gender**	
Female	350
Male	51
Non-binary	2
**Sexual orientation**	
Heterosexual	325
LGBTQ+	72
Prefer not to say	6

This table shows the numbers of responses under the demographic categories of age, disability status, ethnicity, gender, and sexual orientation (*n* = 403).

### 3.2. Discriminating behavior or comments

**Figure 1:**
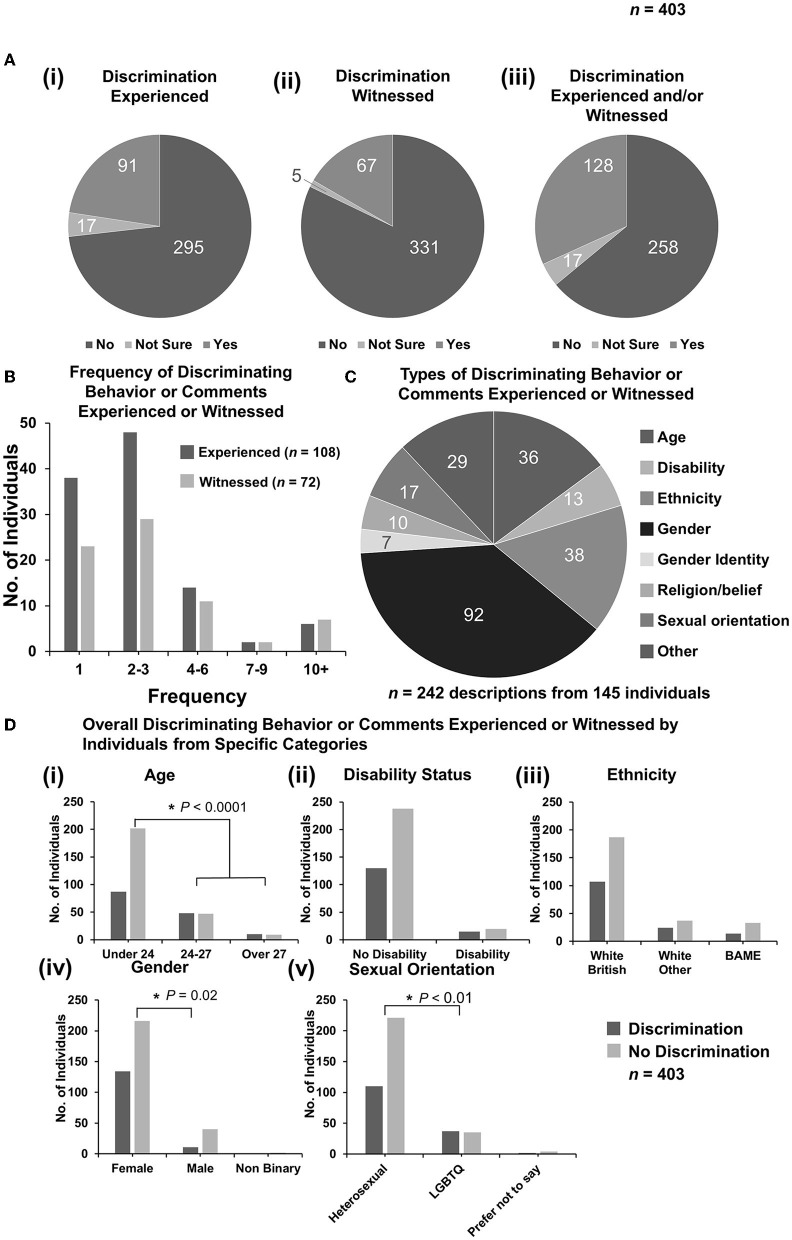
Discriminating behavior or comments experienced or witnessed by respondents. **(A)** Pie charts to demonstrate the numbers of responses for categories of “yes”, “not sure”, and “no” for (i) experienced, (ii) witnessed, or (iii) either experienced or witnessed (*n* = 403). Respondents who both experienced and witnessed discrimination were only counted once. Analysis of free-text descriptions of incidents experienced or witnessed enabled the inclusion of responses of “not sure” to be included as “yes” for further figure parts. **(B)** Column graph to demonstrate the frequency of discriminatory behavior or comments experienced (*n* = 108) or witnessed (*n* = 72) by the number of respondents selecting a frequency category. **(C)** Pie chart demonstrating breakdown of experienced or witnessed discrimination [from **(A)**iii] by category. Some incidents described by respondents could be categorized under more than one category, for example, age and gender (*n* = 242 descriptions from 145 responses). [**(D)**i–v] Bar graphs demonstrating total overall discrimination experienced or witnessed vs. no discrimination broken down by demographic categories. Statistical significance was determined by Pearson's chi-squared test. ^*^P < 0.05.

To address the first aim of the study (to understand discrimination experienced by veterinary students), respondents were asked whether they had personally experienced any discriminating behavior or comments with simple yes, no, or not sure selections. Of the 403 responses included, 295 did not experience any discriminating behavior or comments (73.2%, 95% CI: 68.6–77.5), 17 were unsure, and 91 responded yes giving a total of 108 who were asked for further details (26.8%, 95% CI: 22.5–31.4) [Figure 1A(i)]. To understand the characteristics that discriminatory behavior or comments were targeted against, any participants who responded yes or not sure were asked to select the number of incidents they had experienced or witnessed and select the categories they believed the discrimination fell under. They were then asked to describe the events in a free-text box. Respondents were also asked if they witnessed any discriminatory behavior or comments against another individual with similar further questions to determine the frequency, category, and description of the event(s). Fewer respondents overall witnessed discriminatory behavior or comments with 331 reporting “no” (82.1%, 95% CI: 78.0–85.8), 5 “unsure”, and 67 “yes” giving a total of 72 reports (17.9%, 95% CI: 14.3–22.0) [Figure 1A(ii)]. Respondents fell into four categories, those who had neither experienced nor witnessed any discriminatory behavior or comments, those who had only experienced discriminatory behavior or comments, those who had only witnessed discriminatory behavior or comments, and those who had both experienced and witnessed discriminatory behavior or comments. For the analysis presented, those experiencing and those witnessing were combined while ensuring any reporting of both experienced and witnessed events was not double counted. When combined, 258 respondents did not experience or witness any discriminatory behavior or comments (64.0%, 95% CI: 59.1–68.7), 17 were “unsure”, and 128 had either experienced or witnessed discriminatory behavior or comments giving a total of 145 reports (36.0, 95% CI: 31.3–40.9) [Figure 1A(iii)].

Respondents who responded with “not sure” and had described the events were included in the “yes” category for further analysis (*n* = 108 experienced, *n* = 72 witnessed, *n* = 145 combined). Most respondents experienced or witnessed 1 or 2–3 events (Figure 1B). A proportion of respondents selected more than one category for the described events giving 242 category selections under the 145 individuals (Figure 1C). The most frequent category reported was gender-based discrimination with 92 selections (38.0%, 95% CI: 31.9–44.5), followed by ethnicity with 38 (15.7%, 95% CI: 11.4–20.9), and age with 36 (14.9%, 95% CI: 10.6–20.0) (Figure 1C). Under “other” were descriptions of body shape, professional interests, education, and dietary choices, but primarily socioeconomic status (see Supplementary Table 1). Some descriptions under “other” were determined to be examples of gender discrimination, disability, and ethnicity so were included in analyses as examples under their appropriate categories based on the respondent's description of the event(s).

Responses were grouped based on the demographic characteristics of age, disability status, ethnicity, gender, and sexual orientation. Cross-tabulations were performed under each category to determine if respondents with specific marginalized identities experienced or witnessed more discriminatory behavior or comments overall than respondents within the majority group of that characteristic (Figure 1D). Respondents could select multiple different characteristics and so may have been in the marginalized group for one characteristic and a majority group for another. A chi-squared statistic was determined for each cross-tabulation. Under the category of age, students were grouped as “under 24” (age if a first degree), “24–27” (age if second degree), and “over 27” (age if career choice later in life) years. Analyses were performed where the two younger categories were grouped and compared to the oldest and where the two older categories were grouped and compared to the youngest. When the two older categories were compared with the youngest, the older respondents experienced or witnessed significantly more (50.9%, 95% CI: 41.4–60.4) discriminatory behavior or comments than younger respondents (30.1%, 95% CI: 24.9–35.8), {*X*^2^ [1 d.f., *n* = 289 (under 24) vs. *n* = 114 (over 24)] = 15.32, *p* < 0.001} [Figure 1D(i)]. Individuals with a disability did not experience or witness more total discriminatory behavior or comments than those with no disability {*X*^2^ [1 d.f., *n* = 368 (no disability) vs. *n* = 35 (disability)] = 0.77, *p* > 0.05} [Figure 1D(ii)]. Respondent ethnicity was grouped into white British, white other (including, Irish, continental European, and North American), or BAME (black, Asian, or minority ethnic), with one respondent preferring not to answer and therefore not being included in this analysis. Analysis was performed as both a comparison of white British vs. other ethnicity and white vs. BAME. In both analyses, there was no significant difference in total discrimination experienced or witnessed between different ethnic groups {*X*^2^ [1 d.f., *n* = 294 (white British) vs. *n* = 108 (other ethnicity)] = 0.05, *p* > 0.05; *X*^2^ [1 d.f., *n* = 355 (white) vs. *n* = 47 (BAME)] = 0.91, *p* > 0.05} [Figure 1D(iii)]. By gender, female respondents experienced or witnessed a significantly greater amount of total discrimination of 38.3% (95% CI: 33.2–43.6) compared to 21.6% (95% CI: 11.3–35.3) of male respondents (two respondents were non-binary and did not experience or witness any discrimination) {*X*^2^ [1 d.f., *n* = 350 (female) vs. *n* = 51 (male)] = 5.54, *p* = 0.02} [Figure 1D(iv)]. Finally, respondents were grouped by the response to sexual orientation as either heterosexual or LGBTQ+, with six preferring not to answer the sexual orientation question and not being included in this analysis. Those within the LGBTQ+ group experienced or witnessed significantly more total discrimination than those in the heterosexual group of 51.4% (95% CI: 39.3–63.4) compared to 33.9% (95% CI: 28.7–39.3) {*X*^2^ [1 d.f., *n* = 325 (heterosexual) vs. *n* = 72 (LGBTQ+)] = 8.41, *p* = 0.005} [Figure 1D(v)].

For the qualitative data, responses were coded inductively. For gender-based discrimination, frequent sub-categories included: women not being seen as capable; men being favored for clinical tasks, clients preferring male vets; sexual harassment; assumptions about children/maternity leave; and sexist hiring practices (see Supplementary Table 2). For example:

“*Male farm vet told me I wouldn't make a good farm or mixed vet because I was a woman it meant I was (a) less able to do manual tasks, and (b) would want to have babies which wouldn't suit on call because I couldn't leave a kid home alone…”*

For ethnicity/race-based discrimination, frequent sub-categories included: clients being racist/xenophobic; specific anti-Asian racism; specific anti-Gypsy/Traveler racism; and preferences for white vets (see Supplementary Table 3). Much of this was purportedly due to the perpetrators, including veterinary professionals, airing discriminatory views as a result of the COVID-19 pandemic. For example:

“*They were making racist comments about Chinese people spreading coronavirus and being ‘disgusting.’”*

For age-based discrimination, qualitative responses often referred to youthful appearance and lack of perceived experience, which is likely due to the fact that while the older students are older than the typical first-degree student, they are still in their 20s. Indeed, one comment, in particular, stated that the respondent was accused of having a limited understanding:

“Repeated comments regarding age–being referred to as a ‘kid’ and being told that I have limited understanding of life despite being 27.”

Qualitative responses of respondents who selected the 28–35 age option described discriminatory comments relating to greater age:

“As a mature student, I was told that I was selfish for taking the place of a younger student that would have had a longer career ahead of them. This was said by a farm vet.”

Discrimination on grounds of sexuality was only 7% of the total discrimination encountered by respondents to this survey, which is similar to the 5% and 6% from the Veterinary Voice Survey and BVA discrimination questionnaires, respectively (19, 28). However, the problem of sexual discrimination against veterinary students may be higher than this because as one LGBTQ+ student said:

“*I don't tend to disclose any personal info on EMS placements in case of discrimination, so I haven't experienced any but I think I may.”*

Therefore, there may be less discrimination being experienced as students are not “out” to members of staff at EMS placements. This could be explained by evidence that it is a disclosure of gay/lesbian sexuality or bisexuality that causes higher stress levels in the workplace rather than being closeted to one's colleagues (29).

### 3.3. Discriminatory behavior or comments targeting specific characteristics

**Figure 2:**
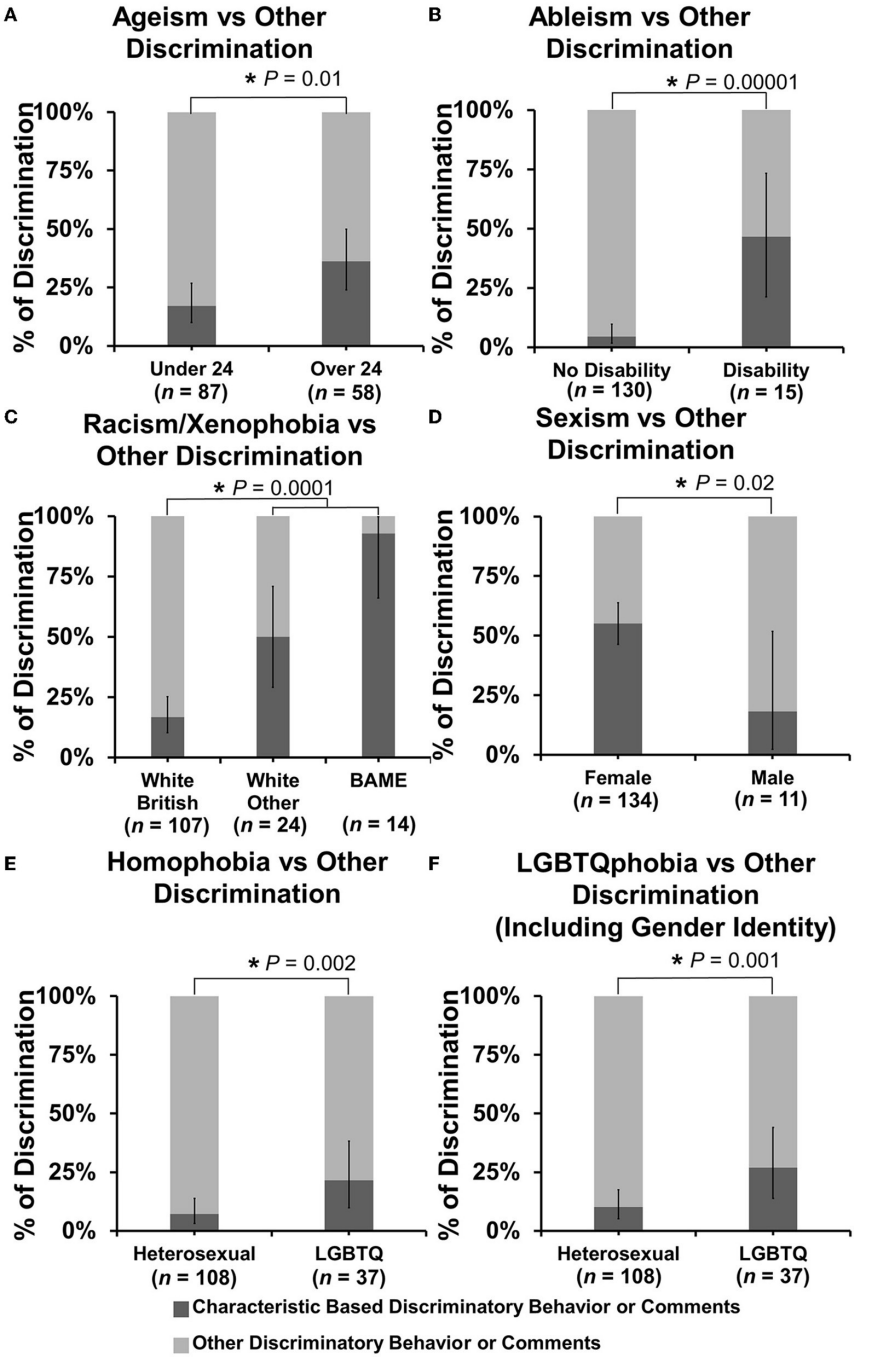
Percentage of discriminating behavior or comments experienced or witnessed targeted toward specific characteristics. Discriminating behavior or comments were categorized based on age (ageism), disability status (ableism), ethnicity (racism/xenophobia), gender/sex (sexism), sexual orientation (homophobia), and LGBTQ status including orientation and gender identity (LGBTQphobia), and plotted as bar charts under relevant respondent demographic categories as percentages vs. any other type of discrimination experienced or witnessed. **(A–F)** Bar charts of percentage proportion of specific discrimination vs other discrimination by respondent demographic. Error bars represent 95% confidence intervals for proportion calculated by the Binomial “Exact” method. Statistical significance was determined by Pearson's chi-squared test of the association of specific discrimination between respondent groups under each category. *P < 0.05.

In exploring the patterns of discrimination experienced by those who reported identifying with one or more marginalized identities further, the authors examined whether these respondents encountered discriminatory behaviors related to their characteristics to a greater degree than those who did not identify as having these same characteristics. Pearson's chi-square tests were used to ascertain if there was a statistically significant association between respondent demographic and the type of discrimination experienced or witnessed. Data are displayed as percentages (Figure 2).

Respondents over 24 years of age experienced or witnessed significantly more discrimination against age than those under 24 years of age at 36.2% (95% CI: 24.0–49.9) of events vs. 17.2% (95% CI: 10.0–26.8) {*X*^2^ [1 d.f., *n* = 87 (under 24) vs. *n* = 58 (over 24)] = 6.71, *p* = 0.0096} (Figure 2A). Respondents with a disability experienced or witnessed significantly more ableism than those with no reported disability at 46.7% (95% CI: 21.3–73.4) of events vs. 4.6% (95% CI: 1.7–9.8) {*X*^2^ [1 d.f., *n* = 130 (no disability) vs. *n* = 15 (disability)] = 18.84, *p* < 0.00001} (Figure 2B). Under the category of racism/xenophobia, the same analysis of comparing white British vs. other ethnicity and white vs. BAME was conducted. Respondents from any other ethnicity than white British experienced or witnessed significantly more racism/xenophobia than white British respondents at 65.8% (95% CI: 48.7–80.4) of events vs 16.8% (95% CI: 10.3–25.3) {*X*^2^ [1 d.f., *n* = 107 (white British) vs. *n* = 38 (other ethnicity)] = 15.18, *p* < 0.0001} and BAME respondents experienced 92.9% (95% CI 66.1–99.8) of events vs. 22.9% (95% CI: 16.0–31.1) {*X*^2^ [1 d.f., *n* = 131 (white) vs. *n* = 14 (BAME)] = 11.42, *p* = 0.0007} (Figure 2C). Under the category of sexism, female respondents experienced or witnessed more gender-based discrimination with 55.2% (95% CI: 46.4–63.8) of events vs. 18.2% (95% CI: 2.3–51.8) for male respondents {*X*^2^ [1 d.f., *n* = 134 (female) vs. *n* = 11 (male)] = 5.59, *p* = 0.018} (Figure 2D). Under the category of homophobia, LGBTQ+ respondents experienced or witnessed significantly more homophobia at 21.6% (95% CI: 9.8–38.2) of events vs. 7.3% (95% CI: 3.2–13.8) of heterosexual respondents {*X*^2^ [1 d.f., *n* = 108 (heterosexual) vs. *n* = 37 (LGBTQ+)] = 9.53, *p* = 0.002} (Figure 2E), which increased to 27.0% (95% CI: 13.8–44.1) of events vs. 10.2% (95% CI: 5.2–17.5) when gender identity-based discrimination (transphobia) was included {*X*^2^ [1 d.f., *n* = 108 (heterosexual) vs. *n* = 37 (LGBTQ+)] = 10.92, *p* = 0.001} (Figure 2F).

### 3.4. Discriminatory behavior or comments by profession sector

**Figure 3:**
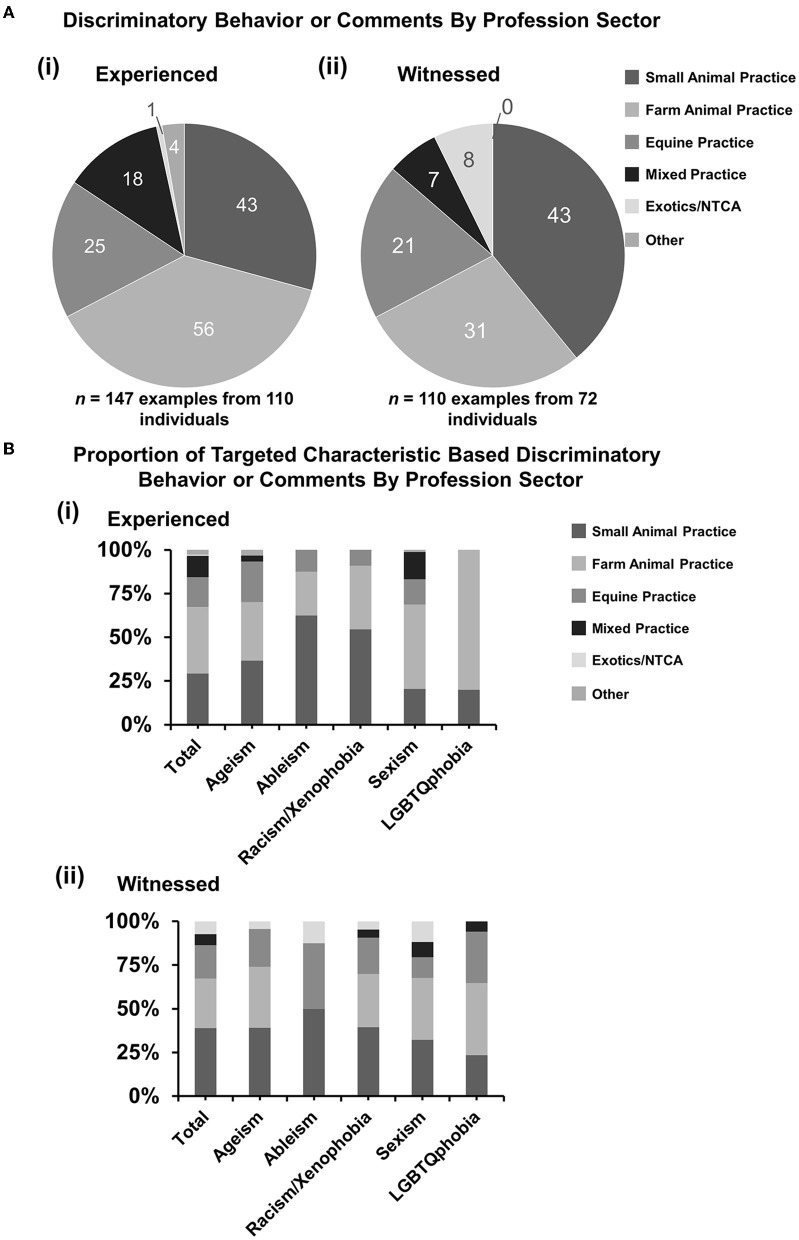
Discriminatory behavior or comments experienced or witnessed by profession sector. Respondents who had responded “yes” or “not sure” were asked questions to select the profession sectors where clinical EMS was undertaken when the discrimination was experienced or witnessed: small animal practice, large animal practice (production animals), equine, mixed practice, exotics and non-traditional companion animals (NTCA), or other. Some respondents had experienced multiple incidents at different EMS placements. [**(A)**i, ii] Pie charts demonstrating a number of selections under profession sector categories for (i) experienced (*n* = 147 examples from 110 individuals) and (ii) witnessed (*n* = 110 examples from 72 individuals) incidents. [**(B)**i, ii] Percentage proportions of examples by discrimination types. Statistical significance was determined by Pearson's chi-squared test of association between total proportions and matching proportions for each category. No statistically significant associations were identified. And 95% confidence intervals have been left off for figure clarity.

For reports of direct experiences of discrimination, 110 individuals made 147 selections, so some individuals had experienced discrimination in more than one EMS placement. For witnessed discrimination, 72 individuals made 110 selections. The majority of discriminatory behavior or comments personally experienced by respondents were while undertaking EMS in farm animal practice with 38.1% (95% CI: 30.2–46.5) of examples reported, with small animal practice coming second with 29.3% (95% CI: 22.1–37.3) [Figure 3A(i)]. For witnessed discriminatory behavior or comments, the majority were experienced in small animal practice with 39.1% (95% CI: 29.9–48.9) reported, with farm animal practice coming second with 28.2% (95% CI: 20.0–37.6). The equine practice was broadly similar for both experienced and witnessed examples with 17.0% (95% CI: 11.3–24.1) and 19.1% (95% CI: 12.2–27.7), respectively.

When broken down by type of discrimination, there were no statistically significant associations between the profession sector and type of discrimination for experienced or witnessed incidents [Figure 3B(i, ii)].

### 3.5. Discriminatory behavior or comments by role of perpetrator

**Figure 4:**
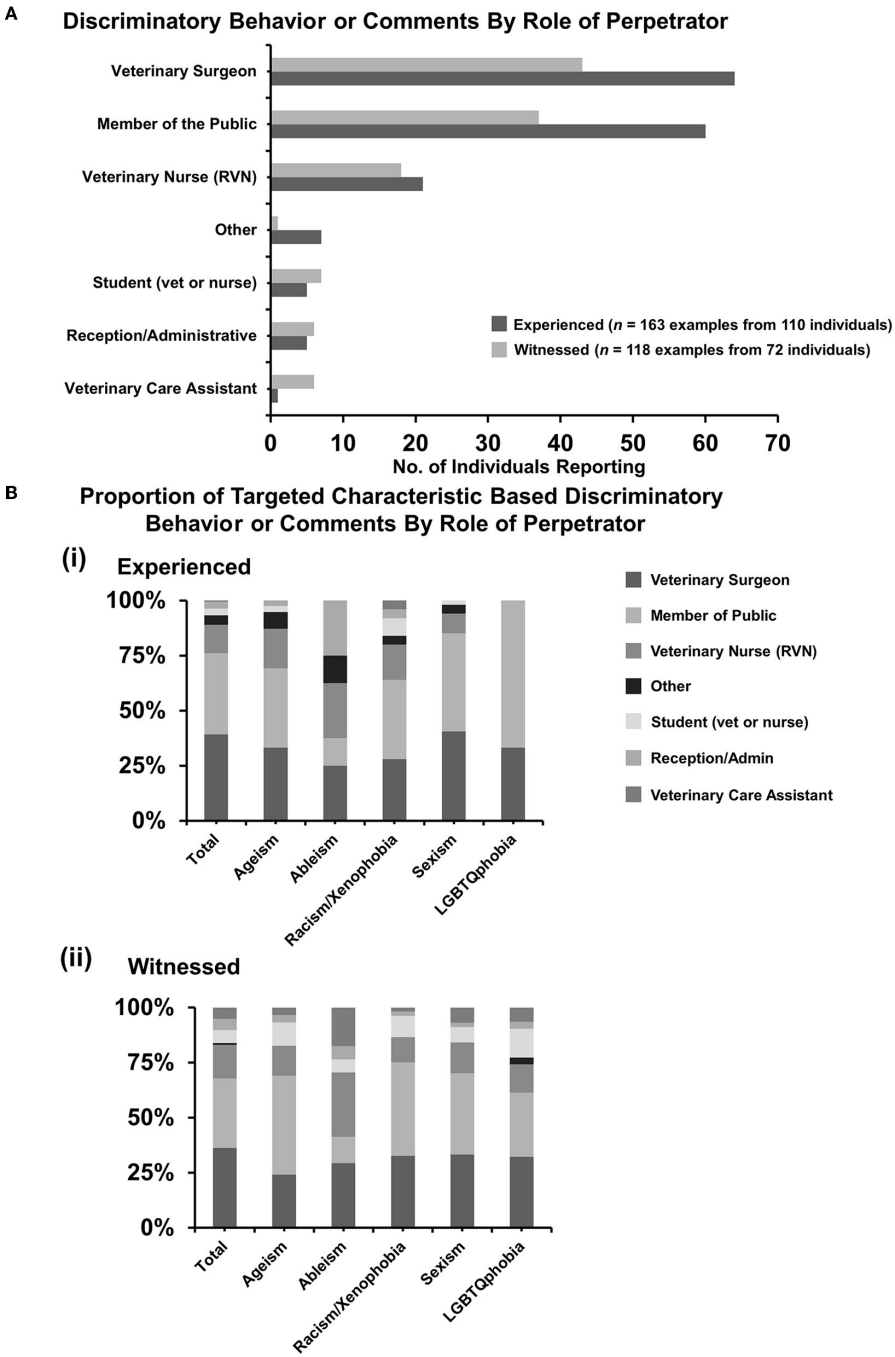
Discriminatory behavior or comments experienced or witnessed by role of perpetrator. Respondents who had responded “yes” or “not sure” were asked questions to select the role(s) of the perpetrator(s) of the discrimination experienced or witnessed. Some respondents had experienced multiple incidents involving sometimes more than one individual in different roles. **(A)** Bar graph demonstrating a number of individuals reporting specific roles of perpetrators for experienced (*n* = 163 examples from 110 individuals) and witnessed (*n* = 118 examples from 72 individuals) discrimination. [**(B)**i, ii] Percentage proportions of examples by discrimination types. Statistical significance was determined by Pearson's chi-squared test of association between total proportions and matching proportions for each category. No statistically significant associations were identified. In addition, 95% confidence intervals have been left off for figure clarity.

For reports of direct experiences of discrimination, the 110 individuals made 163 selections, so some incidents had more than one perpetrator involved. For witnessed discrimination, 72 individuals made 118 selections. Many incidents were from veterinary surgeons with 64 of 163 (39.3%, 95% CI: 31.7–47.2) examples for experiences and 43 of 118 (36.4%, 95% CI: 27.8–45.8) for witnessed discrimination (Figure 4A). Members of the public were second with 60 of 163 (36.8%, 95% CI: 29.4–44.7) examples for experiences and 37 of 118 (31.4%, 95% CI: 23.1–40.5) for witnessed discrimination, respectively (Figure 4A).

Next, the authors wanted to determine if particular types of discriminatory behavior or comments were from people of a particular role as the qualitative free-text descriptions by respondents indicated that sexist comments may be more prevalent in farm practice or racist/xenophobic comments may be more prevalent in small animal practice (Supplementary Tables 2, 3). Pearson's chi-square test revealed that there was no statistically significant association between the type of discriminatory behavior and the role of the perpetrator (Figure 4B).

### 3.6. Respondents' reporting of discriminatory behavior or comments

**Figure 5:**
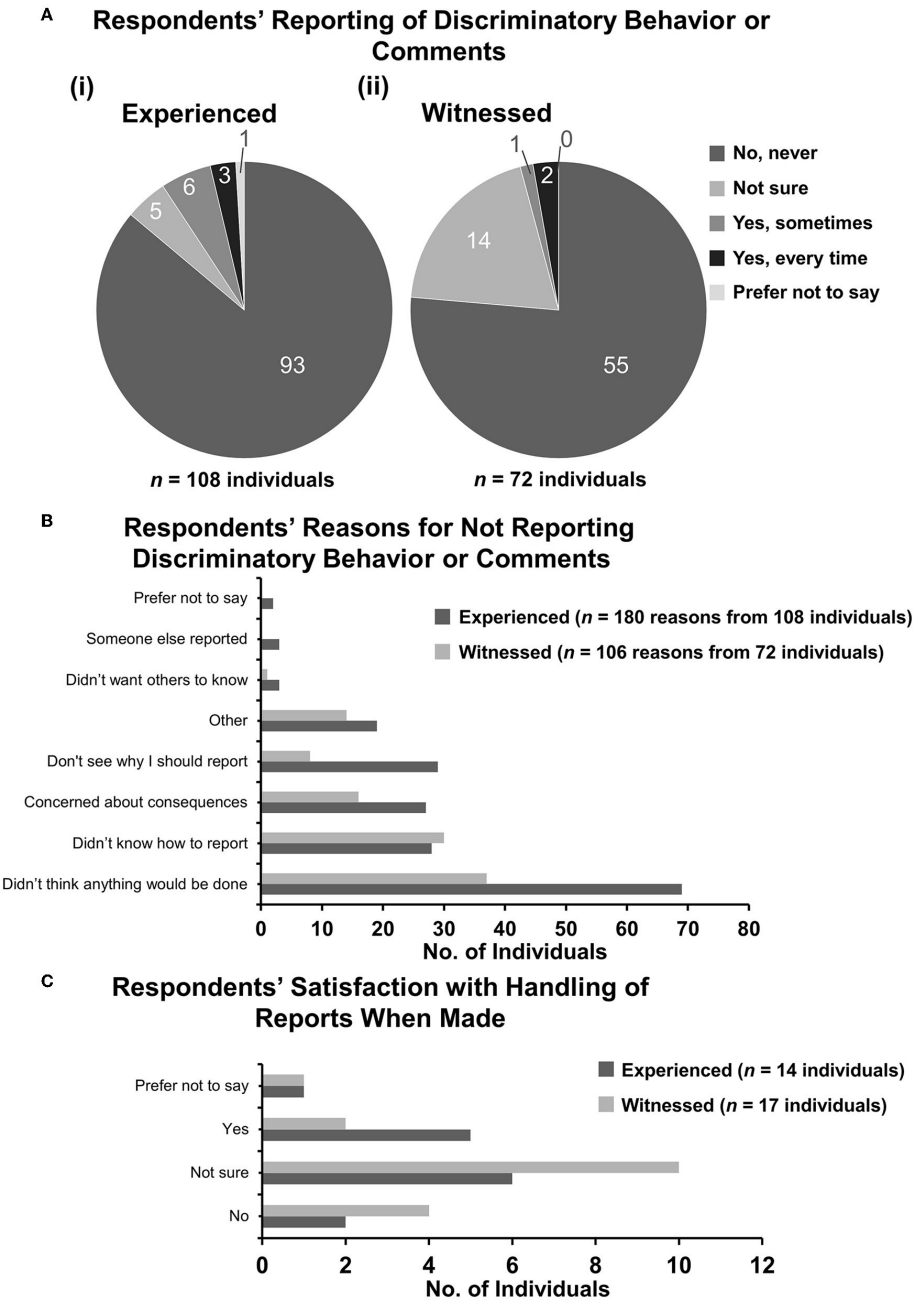
Respondents' reporting of discriminatory behavior of comments. Respondents who had responded “yes” or “not sure” were asked whether they had reported the incident(s) experienced or witnessed to anyone. [**(A)** i, ii] Pie charts demonstrating the number of respondents selecting each category of reporting for (i) experienced and (ii) witnessed incidents. **(B)** Bar graph demonstrating reasons selected by respondents who did not report incidents to anyone else. Some respondents had several reasons. **(C)** Bar graph demonstrating satisfaction of handling of reports when reports were made.

Findings from the BVA Discrimination Survey showed that only two-thirds of discriminatory incidents are reported (19). The third aim of the current study was to identify whether respondents had reported incidents experienced or witnessed and seek further details of possible reasons behind the decision of whether to report. In this question, of the 108 respondents who answered, 86.1% (95% CI: 78.1–92.0) of discriminatory behavior or comments experienced went unreported [Figure 5A(i)] and 76.4% (95% CI: 64.9–85.6) of incidents from 72 respondents who witnessed those incidents went unreported [Figure 5A(ii)].

For the incidents not reported, respondents were asked to select the reasons why they did not report. For experienced incidents, the 108 respondents who answered the question selected 180 options, and for witnessed incidents, 72 respondents selected 106 options. The majority option for both experienced and witnessed incidents was “Didn't think anything would be done” at 38.3% (95% CI: 31.2–45.9) and 34.9% (95% CI: 25.9–44.8), respectively. “Didn't know how to report” was a clear second option for witnessed incidents at 28.3% (95% CI: 20.0–37.9) (Figure 5B). For experienced incidents, “Don't see why I should report”, “Didn't know how to report”, and “Concerned about consequences” were all broadly similar at 16.1% (95% CI: 11.1–22.3), 15.6% (95% CI: 10.6–21.7), and 15.0% (95% CI: 10.1–21.1), respectively.

Rates of confirmed reports were very low, only nine respondents who experienced incidents and only three who witnessed incidents reported them (Figure 5A). The extent to which respondents were satisfied with the outcomes of the reports varied (Figure 5C).

Supplementary Table 4 shows respondents' qualitative responses when they selected “other” as the reason why the incident was not reported. The most frequent coded reasons for this was that they feel the discrimination incidents were not important enough to report. Indeed it has been discussed that it is often small and subtle incidents termed “microaggressions” as well as indirect expressions of prejudice that can significantly contribute to the overall problems of discrimination experienced and witnessed within the medical profession (30). Following this, other reasons included: feeling unable to speak up due to their position as an EMS student; being concerned about how they would be perceived if they reported; and fearing it may have implications on their EMS requirements. For example:

“Because it is so crucial to undertake a vast amount of EMS I just feel like I should shut up and deal with it…”

Similarly, in the BVA Discrimination Survey, the most common response was ignoring the incident with reasons cited as being something respondents just had to put up with (19).

### 3.7. Respondent attitudes

**Figure 6:**
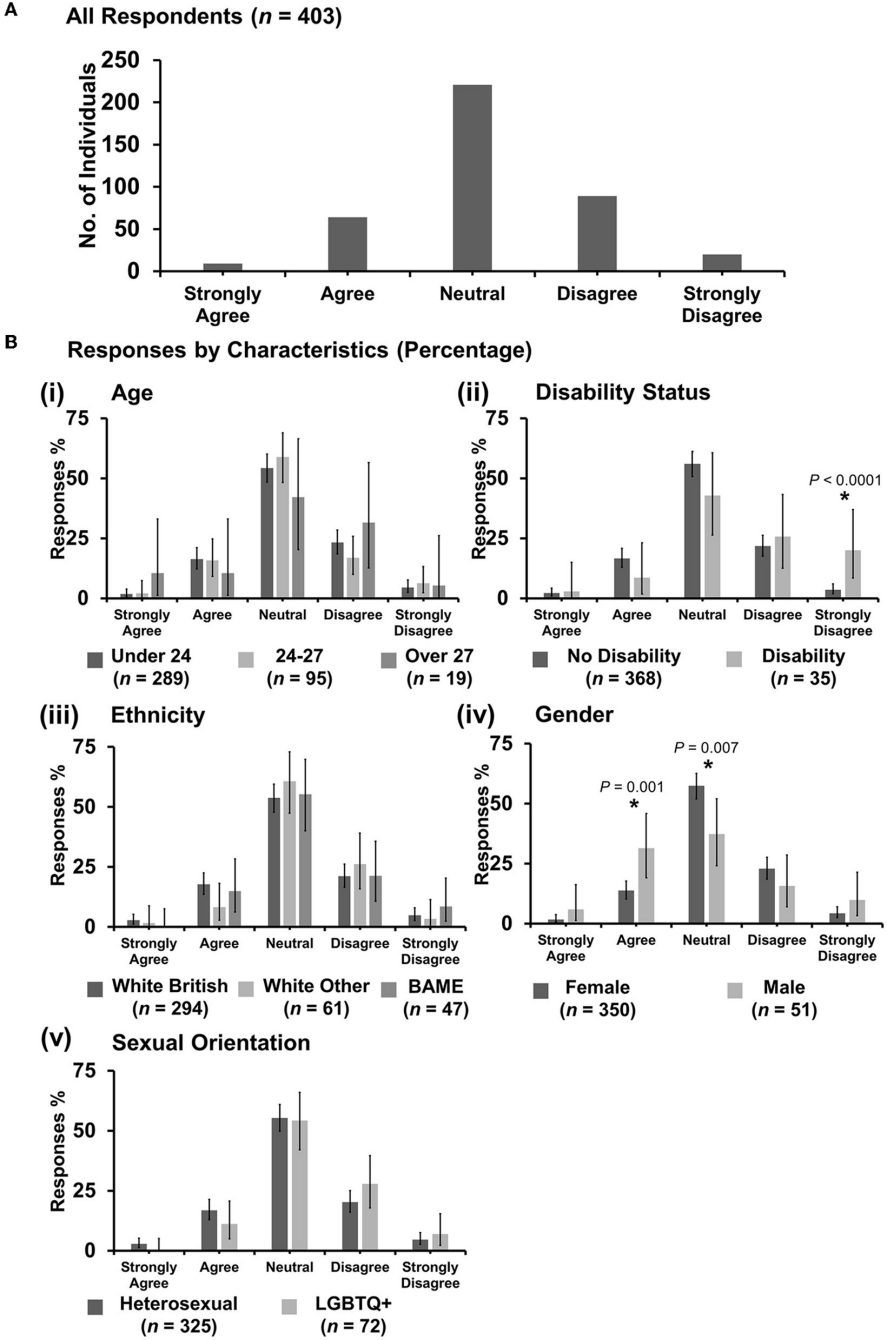
Likert scale selections for the statement: professional bodies such as RCVS/BVA/AVS/Vet schools are doing enough to tackle discrimination. **(A)** Bar graph demonstrating the number of respondents selecting each Likert scale response. [**(B)**i–v] Bar graphs demonstrating by percentage proportion the number of responses in each Likert category by the group under the demographic categories of age, disability status, ethnicity, gender, and sexual orientation. Error bars represent 95% confidence intervals calculated by the Binomial “Exact” method. Statistical significance was determined by Pearson's chi-squared test of association between respondent's group under category and Likert scale selection. *P < 0.05.

**Figure 7:**
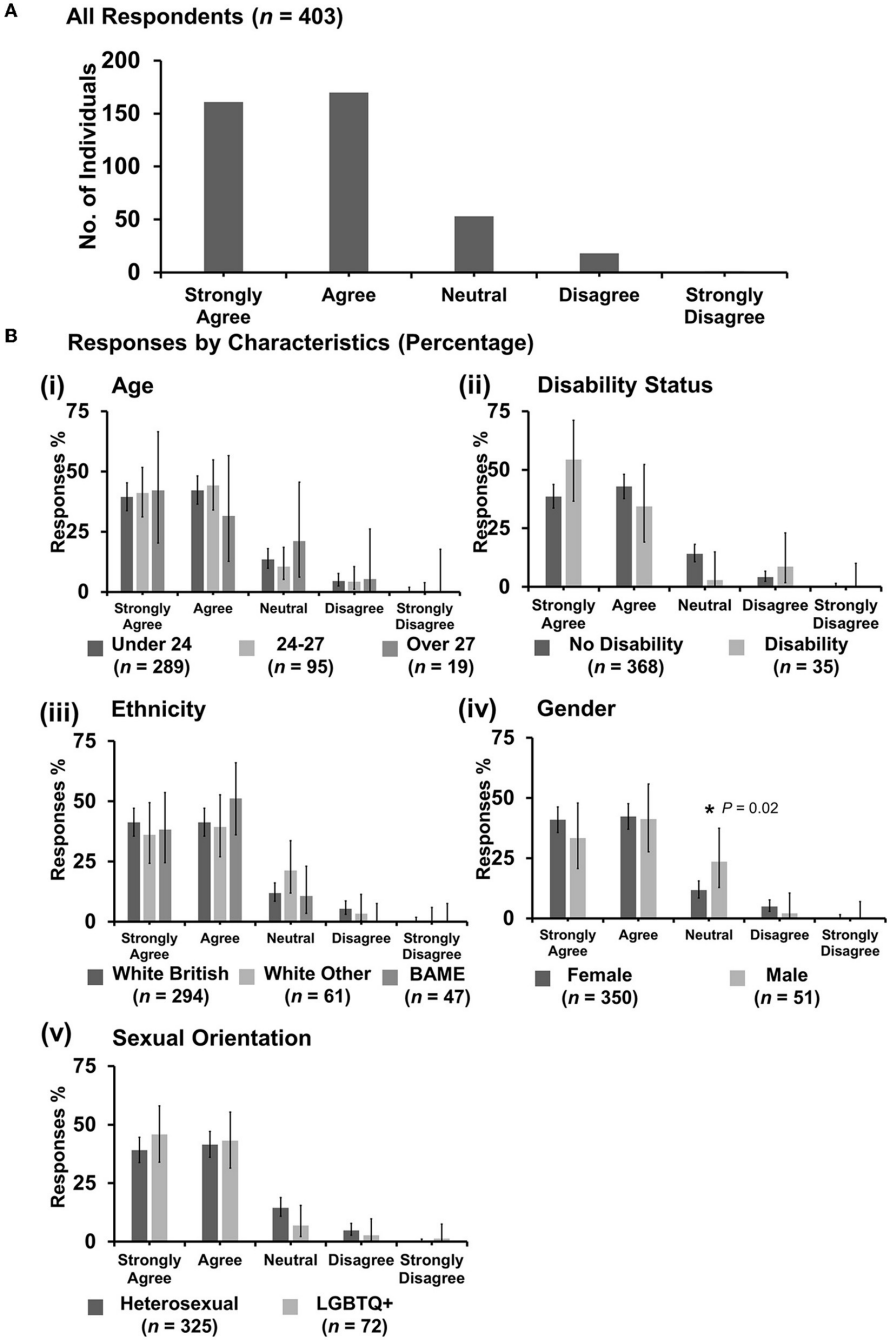
Likert scale selections for the statement: discrimination against veterinary students is an important issue. **(A)** Bar graph demonstrating the number of respondents selecting each Likert scale response. [**(B)**i–v] Bar graphs demonstrating by percentage proportion the number of responses in each Likert category by the group under the demographic categories of age, disability status, ethnicity, gender, and sexual orientation. Error bars represent 95% confidence intervals calculated by the Binomial “Exact” method. Statistical significance was determined by Pearson's chi-squared test of association between respondent's group under category and Likert scale selection. *P < 0.05.

**Figure 8:**
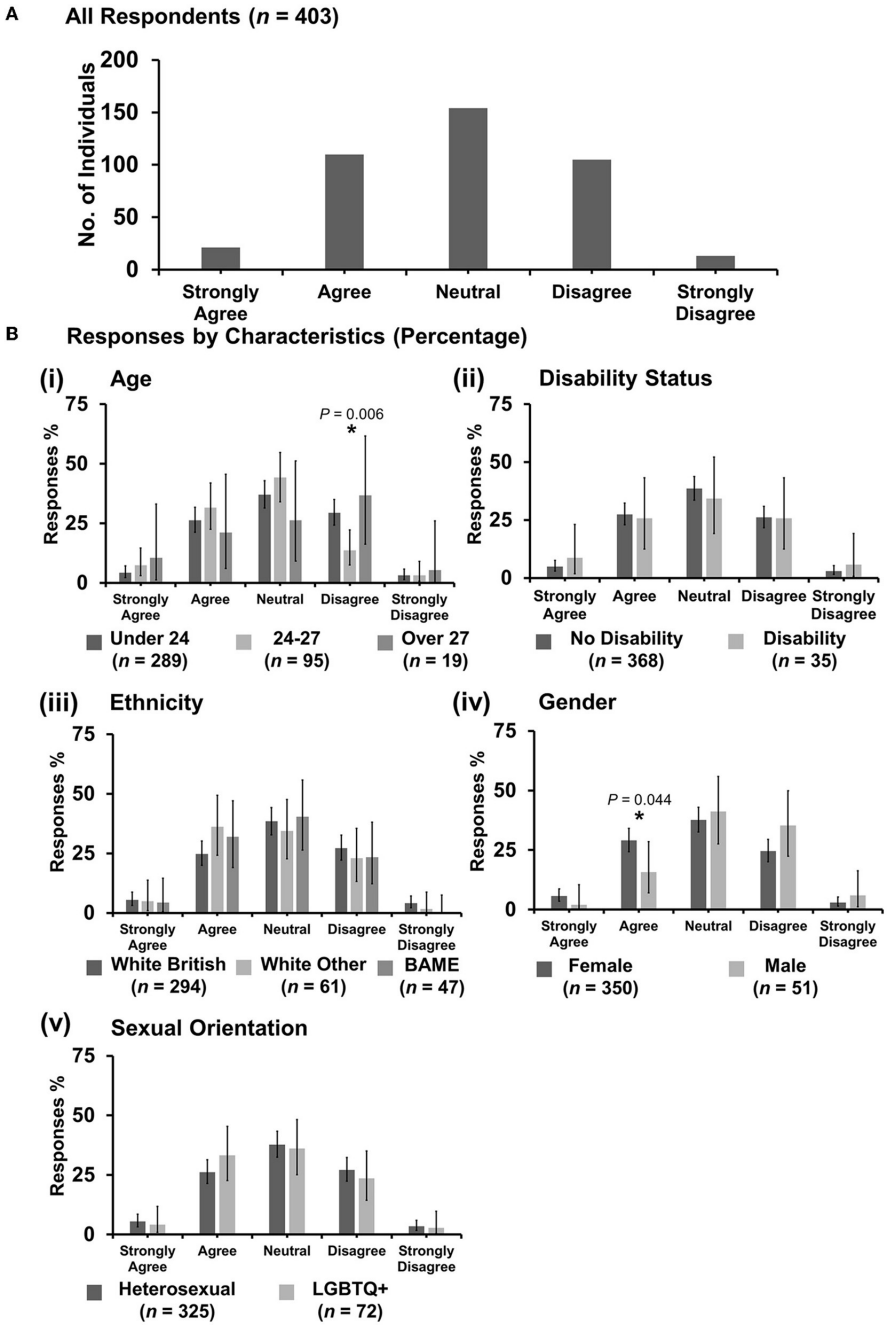
Likert scale selections for the statement: discrimination against veterinary students happens frequently. **(A)** Bar graph demonstrating the number of respondents selecting each Likert scale response. [**(B)**i–v] Bar graphs demonstrating by percentage proportion the number of responses in each Likert category by the group under the demographic categories of age, disability status, ethnicity, gender, and sexual orientation. Error bars represent 95% confidence intervals calculated by the Binomial “Exact” method. Statistical significance was determined by Pearson's chi-squared test of association between respondent's group under category and Likert scale selection. *P < 0.05.

**Figure 9:**
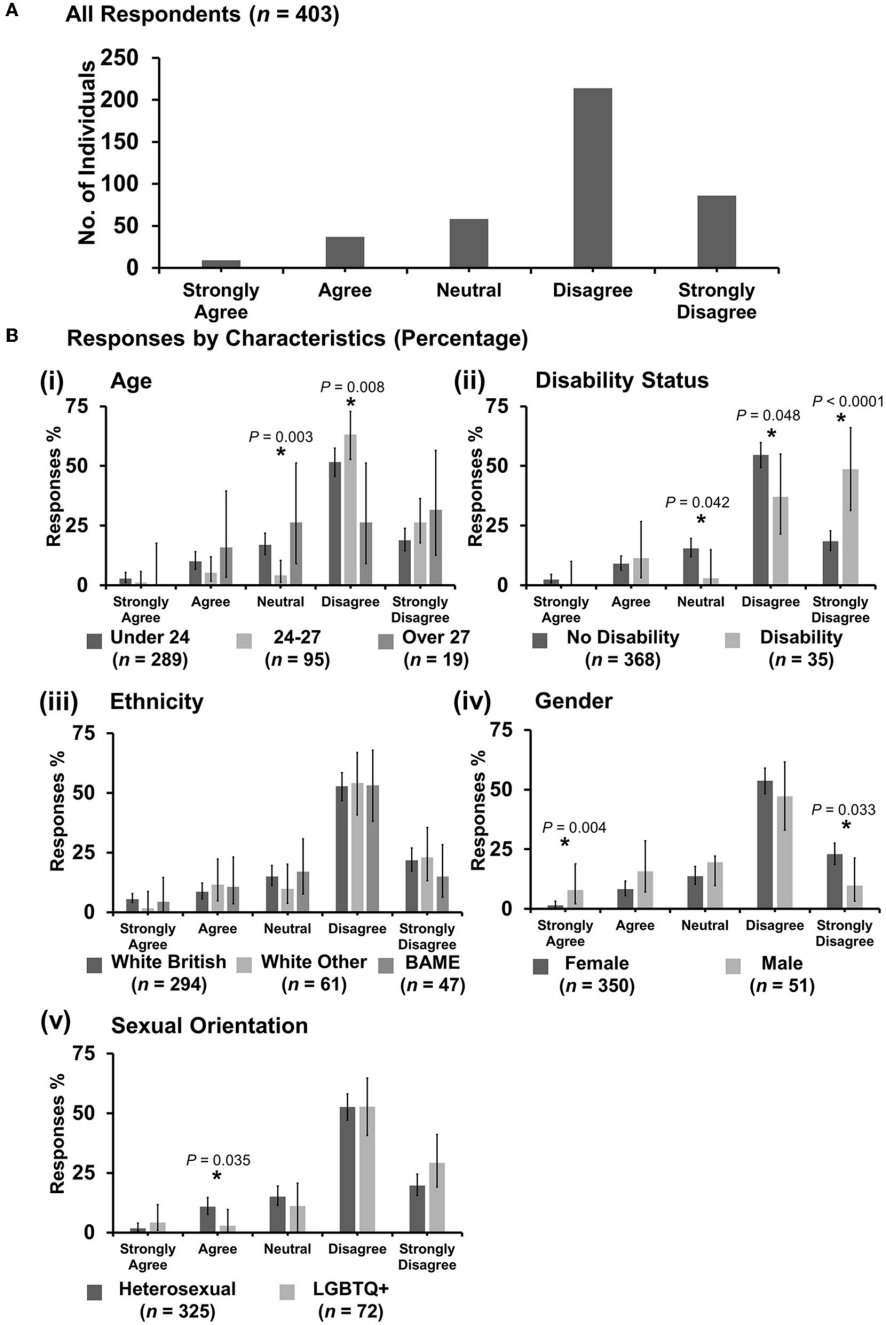
Likert scale selections for the statement: discrimination against female veterinary students is no longer an issue due to being a majority. **(A)** Bar graph demonstrating the number of respondents selecting each Likert scale response. [**(B)**i–v] Bar graphs demonstrating by percentage proportion the number of responses in each Likert category by the group under the demographic categories of age, disability status, ethnicity, gender, and sexual orientation. Error bars represent 95% confidence intervals calculated by the Binomial “Exact” method. Statistical significance was determined by Pearson's chi-squared test of association between respondent's group under category and Likert scale selection. *P < 0.05.

**Figure 10:**
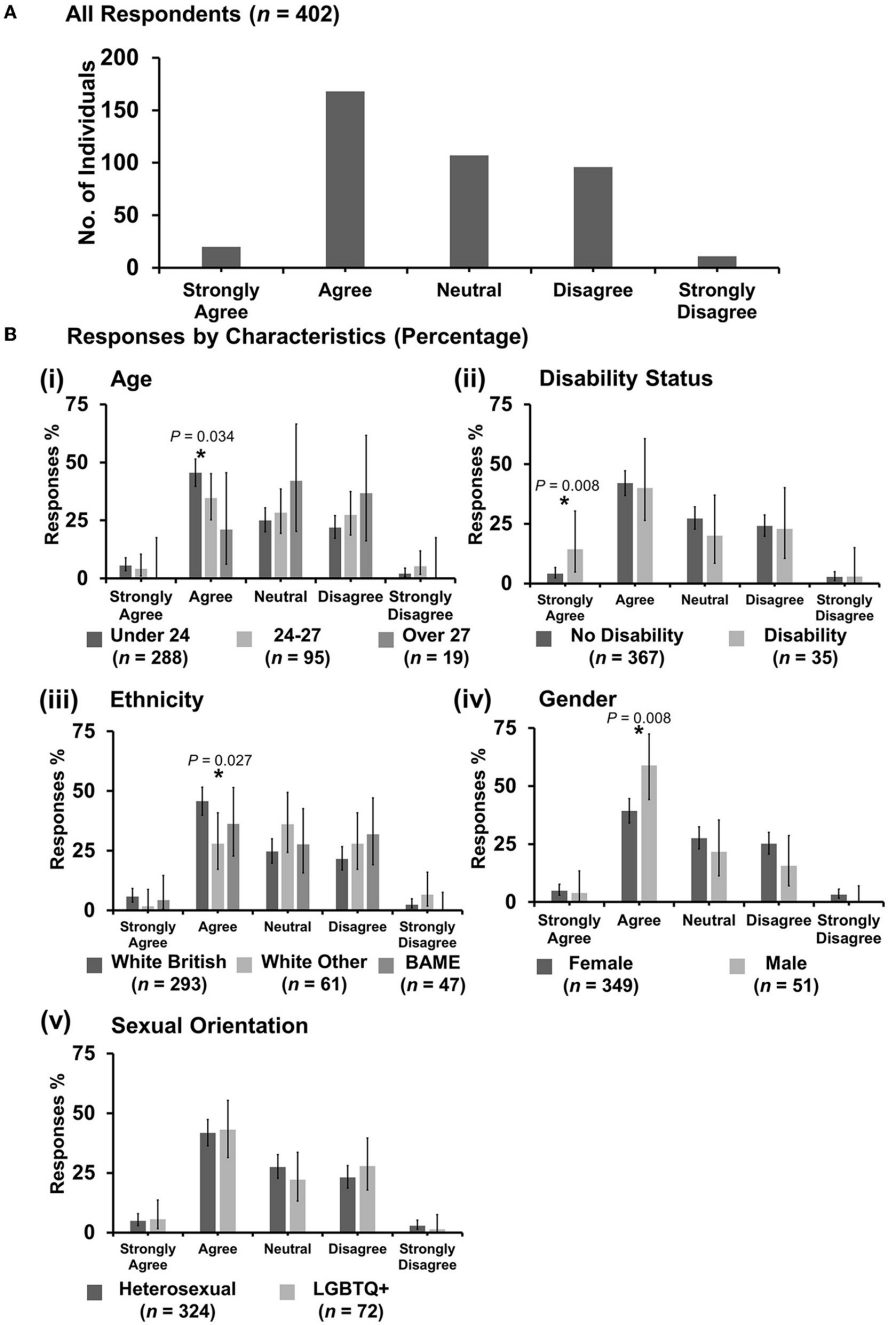
Likert scale selections for the statement: older veterinary students are discriminated against less. **(A)** Bar graph demonstrating the number of respondents selecting each Likert scale response. [**(B)**i–v] Bar graphs demonstrating by percentage proportion the number of responses in each Likert category by the group under the demographic categories of age, disability status, ethnicity, gender, and sexual orientation. Error bars represent 95% confidence intervals calculated by the Binomial “Exact” method. Statistical significance was determined by Pearson's chi-squared test of association between respondent's group under category and Likert scale selection. *P < 0.05.

**Figure 11:**
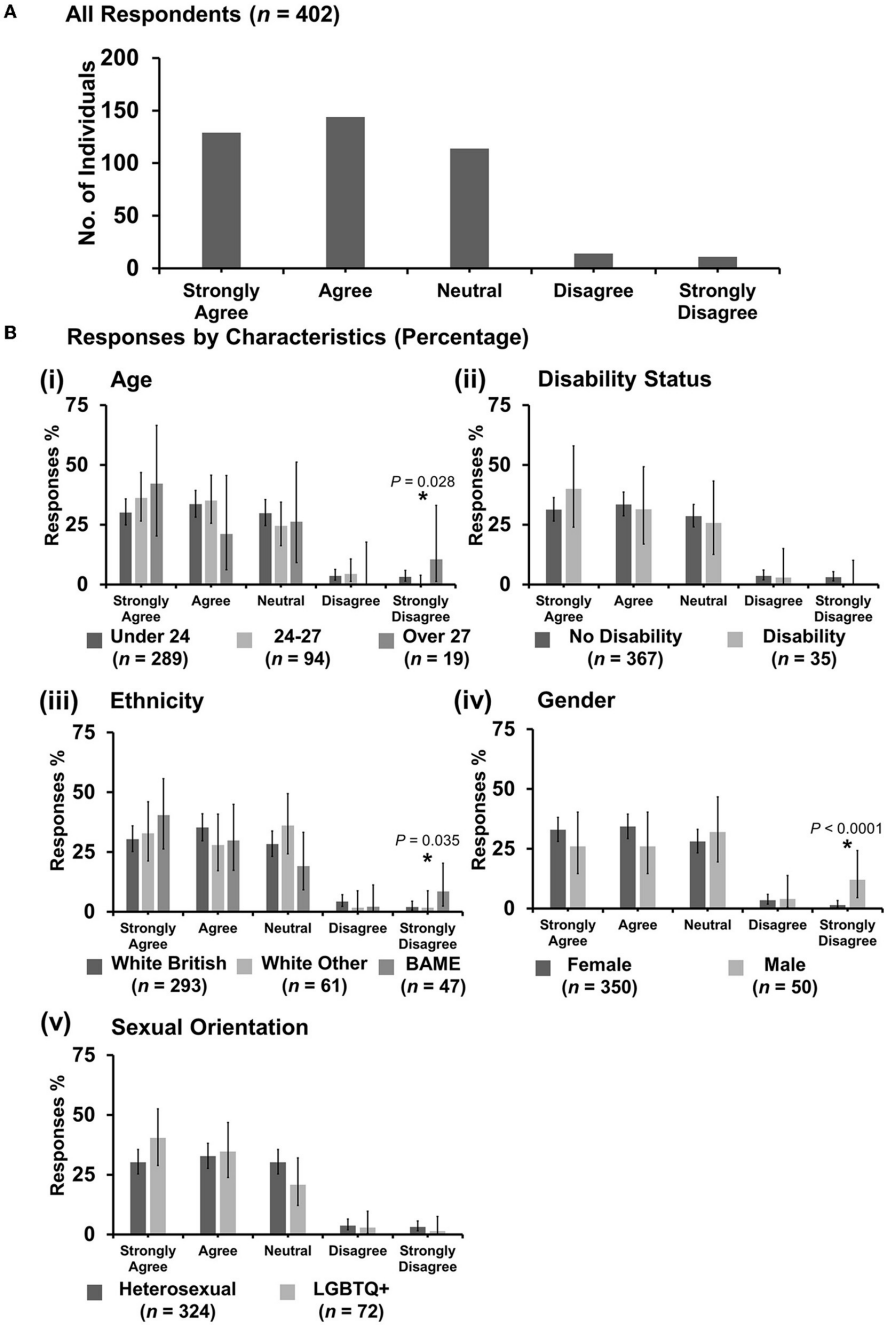
Likert scale selections for the statement: the racial diversity in the veterinary profession needs to be increased. **(A)** Bar graph demonstrating the number of respondents selecting each Likert scale response. [**(B)**i–v] Bar graphs demonstrating by percentage proportion the number of responses in each Likert category by the group under the demographic categories of age, disability status, ethnicity, gender, and sexual orientation. Error bars represent 95% confidence intervals calculated by the Binomial “Exact” method. Statistical significance was determined by Pearson's chi-squared test of association between respondent's group under category and Likert scale selection. *P < 0.05.

**Figure 12:**
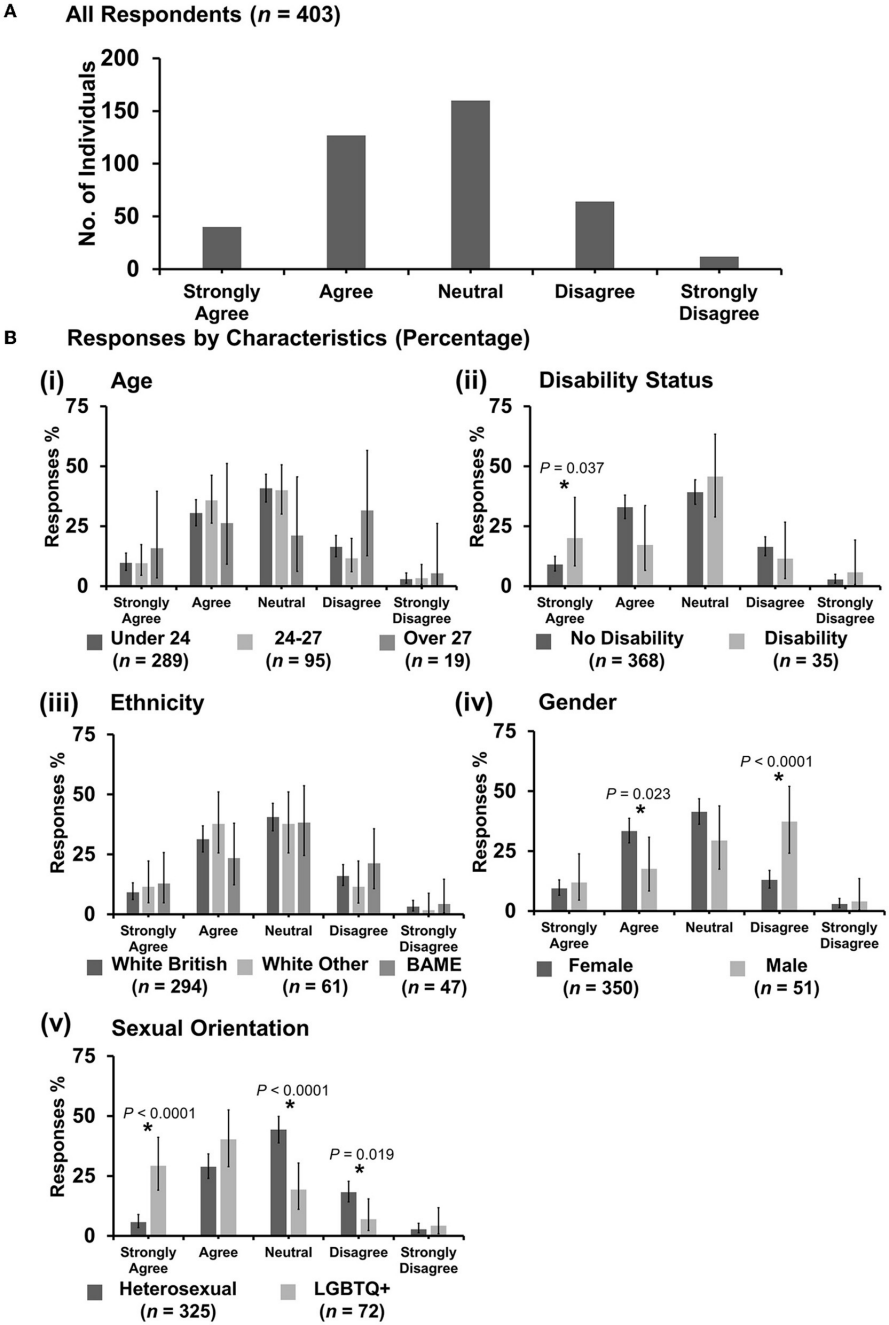
Likert scale selections for the statement: known LGBTQ+ status leads to an increased risk of discrimination for veterinary students. **(A)** Bar graph demonstrating the number of respondents selecting each Likert scale response. [**(B)**i–v] Bar graphs demonstrating by percentage proportion the number of responses in each Likert category by the group under the demographic categories of age, disability status, ethnicity, gender, and sexual orientation. Error bars represent 95% confidence intervals calculated by the Binomial “Exact” method. Statistical significance was determined by Pearson's chi-squared test of association between respondent's group under category and Likert scale selection. *P < 0.05.

**Figure 13:**
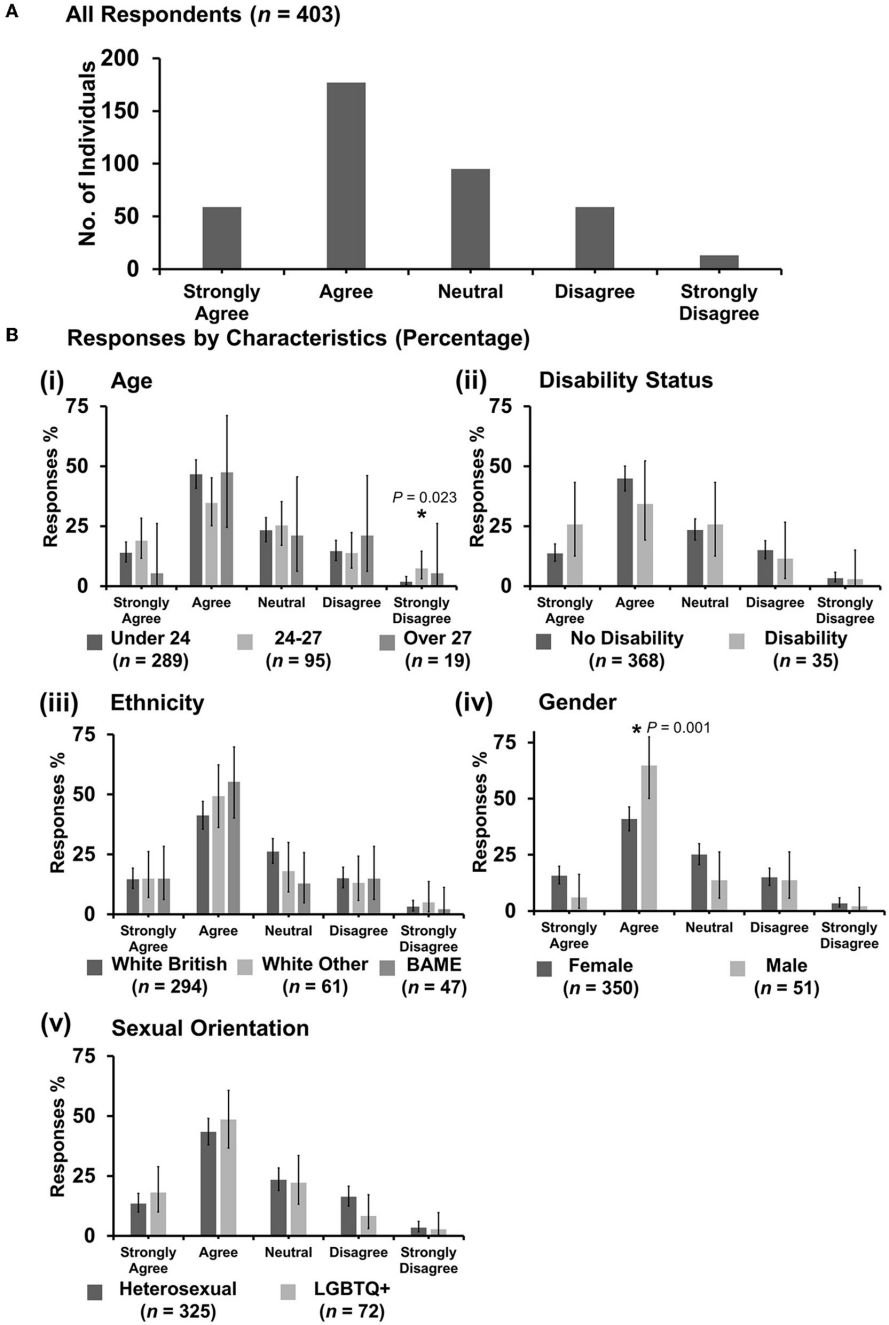
Likert scale selections for the statement: veterinary students are more vulnerable to discrimination than qualified veterinary surgeons/veterinary professionals. **(A)** Bar graph demonstrating the number of respondents selecting each Likert scale response. [**(B)**i–v] Bar graphs demonstrating by percentage proportion the number of responses in each Likert category by the group under the demographic categories of age, disability status, ethnicity, gender, and sexual orientation. Error bars represent 95% confidence intervals calculated by the Binomial “Exact” method. Statistical significance was determined by Pearson's chi-squared test of association between respondent's group under category and Likert scale selection. *P < 0.05.

**Figure 14:**
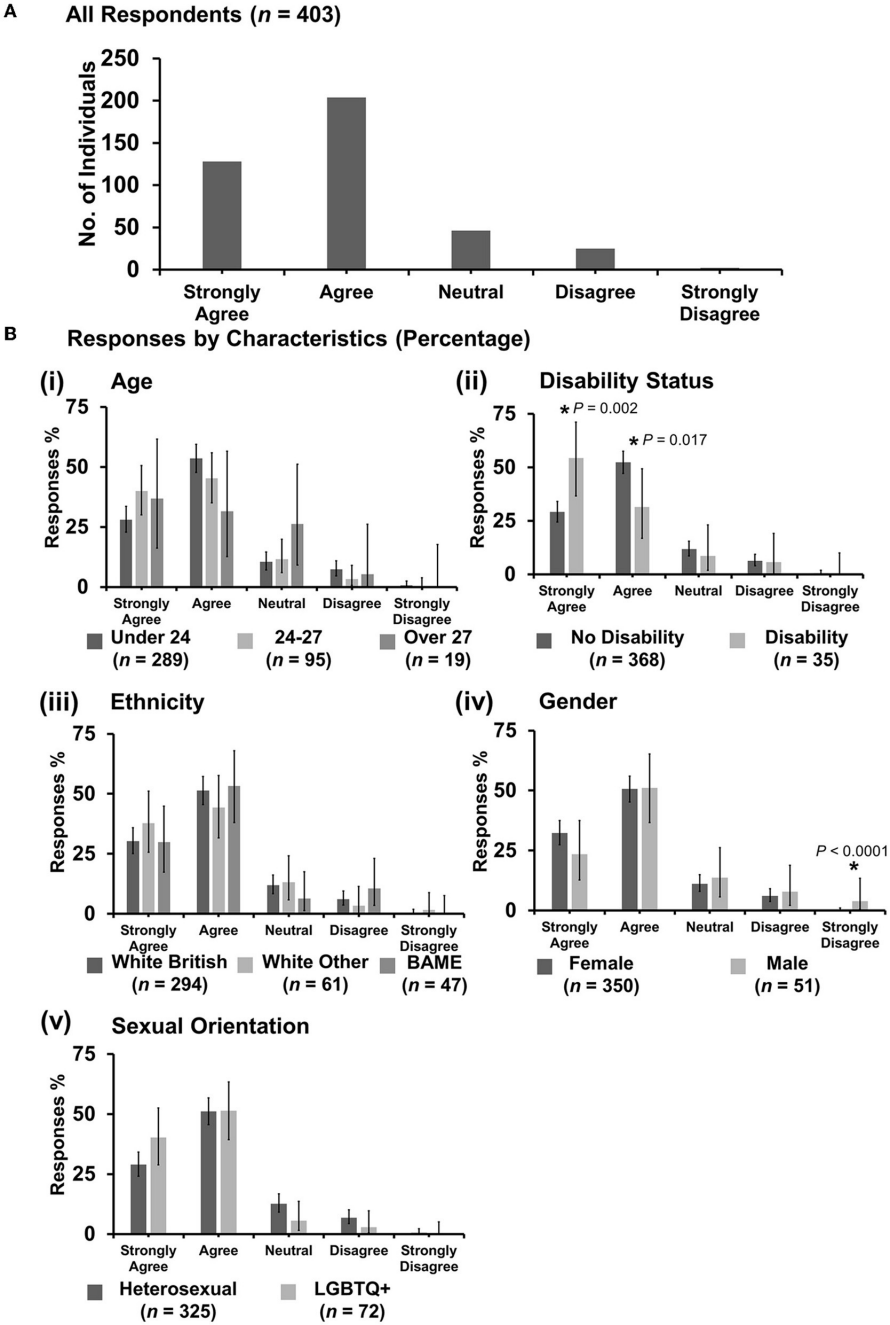
Likert scale selections for the statement: veterinary students are less likely to report discrimination due to fear of consequences. **(A)** Bar graph demonstrating the number of respondents selecting each Likert scale response. [**(B)**i–v] Bar graphs demonstrating by percentage proportion the number of responses in each Likert category by the group under the demographic categories of age, disability status, ethnicity, gender, and sexual orientation. Error bars represent 95% confidence intervals calculated by the Binomial “Exact” method. Statistical significance was determined by Pearson's chi-squared test of association between respondent's group under category and Likert scale selection. *P < 0.05.

To determine student perceptions and attitudes about discrimination in the vet profession, respondents were asked to rate agreement with statements about discrimination while undertaking clinical EMS using the 5-point Likert scale from Strongly Agree to Strongly Disagree. Data are displayed for each statement (Figures 6–**14**) as the number of individuals giving each answer (Figures 6–**14A**) and proportions of responses based on respondent demographics [Figures 6–**14B**(i–v)] as the intersectional experiences of students will vary depending on the group(s) to which they identify and the topic the statement relates to. Pearson's *X*^2^ tests were conducted under each statement testing for correlation between the demographics of respondents as reported in Figure 2, and the Likert score those respondents gave.

#### 3.7.1. “Professional bodies such as RCVS/BVA/AVS/Vet schools are doing enough to tackle discrimination”

Most respondents (221 out of 403) were neutral with an even spread in agreement or disagreement (Figure 6A). However, *X*^2^ tests revealed that respondents reporting as having a disability were more likely to strongly disagree with the statement {*X*^2^ [1 d.f., *n* = 368 (no disability) vs. *n* = 35 (disability)] = 18.38, *p* < 0.0001} [Figure 6B(ii)]. Female respondents were more likely to be neutral {*X*^2^ [1 d.f., *n* = 350 (female) vs. *n* = 51 (male)] = 7.32, *p* = 0.007}, compared to male respondents who were more likely to agree {*X*^2^ [1 d.f., *n* = 350 (female) vs. *n* = 51 (male)] = 10.35, *p* = 0.001} [Figure 6B(iv)]. No other statistically significant associations were identified.

#### 3.7.2. “Discrimination against veterinary students is an important issue”

Most respondents (331 out of 403) agreed (Figure 7A). However, *X*^2^ tests revealed that male respondents were more likely to be neutral with the statement {*X*^2^ [1 d.f., *n* = 350 (female) vs. *n* = 51 (male)] = 5.42, *p* = 0.02} than female respondents [Figure 7B(iv)]. No other statistically significant associations were identified.

#### 3.7.3. “Discrimination against veterinary students happens frequently”

Most respondents (154 out of 403) were neutral with an even spread in agreement or disagreement (Figure 8A). However, *X*^2^ tests revealed that respondents between the ages of 24 and 27 years were more likely to disagree with the statement {*X*^2^ [2 d.f., n = 289 (under 24) vs. *n* = 95 (24–27) vs. *n* = 19 (over 27)] = 10.38, p = 0.006} [Figure 8B(i)] compared to respondents under 24 or over 27 years. Under gender, female respondents were more likely to agree {*X*^2^ [1 d.f., n = 350 (female) vs. n = 51 (male)] = 4.05, p = 0.044} [Figure 8B(iv)] compared to male respondents. No other statistically significant associations were identified.

#### 3.7.4. “Discrimination against female veterinary students is no longer an issue due to being a majority”

Most respondents (214 out of 403) disagreed with the statement (Figure 9A). *X*^2^ tests showed that respondents in the age category 24–27 years were less likely to be neutral than either under 24 or over 27 years {*X*^2^ [2 d.f., *n* = 289 (under 24) vs. *n* = 95 (24–27) vs. *n* = 19 (over 27)] = 11.73, *p* = 0.006}, and respondents over 27 years were less likely to disagree {*X*^2^ [2 d.f., *n* = 289 (Under 24) vs. *n* = 95 (24–27) vs. *n* = 19 (over 27)] = 9.61, *p* = 0.008} [Figure 9B(i)] compared to respondents in categories under 27 years. For data analyzed by disability status, respondents reporting no disability were more likely than those with a disability to respond either neutral {*X*^2^ [1 d.f., *n* = 368 (no disability) vs. *n* = 35 (disability)] = 4.14, *p* = 0.042} or disagree {*X*^2^ [1 d.f., *n* = 368 (no disability) vs. *n* = 35 (disability)] = 3.92, *p* = 0.048} [Figure 9B(ii)]. Respondents with a disability were more likely to strongly disagree {*X*^2^ [1 d.f., *n* = 368 (no disability) vs. *n* = 35 (disability)] = 17.39, *p* < 0.0001} [Figure 9B(ii)}. Further cross-tabulation to check the association between disability status and gender showed no statistical significance (*p* = 0.20). Under gender, female respondents were more likely to strongly disagree {*X*^2^ [1 d.f., *n* = 350 (female) vs. *n* = 51 (male)] = 4.54, *p* = 0.033} compared to male respondents who were more likely to strongly agree {*X*^2^ [1 d.f., *n* = 350 (female) vs. *n* = 51 (male)] = 8.35, *p* = 0.004} [Figure 9B(iv)]. Under sexual orientation, respondents who identified as heterosexual were more likely to agree than those identifying as LGBTQ+ {*X*^2^ [1 d.f., *n* = 325 (heterosexual) vs. *n* = 72 (LGBTQ+)] = 4.45, *p* = 0.035} [Figure 9B(v)]. No other statistically significant associations were identified.

#### 3.7.5. “Older veterinary students are discriminated against less”

Most respondents (168 out of 402) agreed with the statement, with broadly similar numbers either being neutral (107) or disagreeing (96) (Figure 10A). Respondents aged under 24 years were more likely to agree with the statement than older respondents {*X*^2^ [2 d.f., *n* = 288 (under 24) vs. *n* = 95 (24–27) vs. *n* = 19 (over 27)] = 6.79, *p* = 0.034} [Figure 10B(i)]. Respondents with a disability were more likely to strongly agree than those with no disability {*X*^2^ [1 d.f., *n* = 367 (no disability) vs. *n* = 35 (disability)] = 7.06, *p* = 0.008} [Figure 10B(ii)]. Under ethnicity, white British respondents were more likely to agree than either white other or BAME respondents {*X*^2^ [2 d.f., *n* = 293 (white British) vs. *n* = 61 (white other) vs. *n* = 47 (BAME)] = 7.20, *p* = 0.027} [Figure 10B(iii)]. Under gender, male respondents were more likely to agree than female respondents {*X*^2^ [1 d.f., *n* = 349 (female) vs. *n* = 51 (male)] = 7.10, *p* = 0.008} [Figure 10B(iv)]. No other statistically significant associations were identified.

#### 3.7.6. “The racial diversity in the veterinary profession needs to be increased”

Very few respondents disagreed with the statement with 144 out of 402 agreeing, 129 strongly agreeing, and 114 neutral (Figure 11A). However, in cross-tabulation analysis, respondents aged over 27 years were more likely than younger respondents to strongly disagree {*X*^2^ [2 d.f., *n* = 289 (under 24) vs. *n* = 94 (24–27) vs. *n* = 19 (over 27)] = 7.18, *p* = 0.028} [Figure 11B(i)]. Interestingly, respondents of BAME origins were also more likely to strongly disagree than either white other or white British respondents {*X*^2^ [2 d.f., *n* = 293 (white British) vs. *n* = 61 (white other) vs. *n* = 47 (BAME)] = 6.70, *p* = 0.035} Figure 11B(iii). One of the strongly disagreeing BAME respondents clarified their response qualitatively:

“*In terms of my answer to racial diversity; I believe that those who are more suited for the course should apply, no matter what their racial/sexual/gender status is. If we assign a minimum amount of racial diversity then you're putting potentially better suited vets out of a career to satisfy a ridiculous quota. As a boy, it always sits at the back of my mind If I actually would have gotten in this was a male dominated course.”*

Male respondents were more likely to strongly disagree than female respondents {*X*^2^[1 d.f., *n* = 350 (female) vs. *n* = 50 (male)] = 17.83, *p* < 0.0001} [Figure 11B(iv)]. No other statistically significant associations were identified.

#### 3.7.7. “Known LGBTQ+ status leads to an increased risk of discrimination for veterinary students”

Most respondents were neutral (160 out of 403) with the agreement being the next option (127) (Figure 12A). Respondents with a disability were more likely to strongly agree than those with no disability {*X*^2^ [1 d.f., *n* = 368 (no disability) vs. *n* = 35 (disability)] = 4.35, *p* = 0.037} [Figure 12B(ii)]. Interestingly, there was an association between respondents having a disability and identifying as LGBTQ+ with 21.1% of LGBTQ+ respondents having a disability vs. 5.8% of heterosexual respondents {*X*^2^ [1 d.f., *n* = 368 (no disability) vs. *n* = 35 (disability)] = 16.82, *p* < 0.0001}. Under gender, female respondents were more likely to agree with the statement {*X*^2^ [1 d.f., *n* = 350 (female) vs. *n* = 51 (male)] = 5.15, *p* = 0.023} [Figure 12B(iv)], whereas male respondents were more likely to disagree {*X*^2^ [1 d.f., *n* = 350 (female) vs. *n* = 51 (male)] = 17.76, *p* < 0.0001} [Figure 12B(iv)]. LGBTQ+ respondents were more likely to strongly agree {*X*^2^ [1 d.f., *n* = 325 (heterosexual) vs. *n* = 72 (LGBTQ+)] = 35.38, *p* < 0.0001}, compared to heterosexual respondents who were more likely to either be neutral {*X*^2^ [1 d.f., *n* = 325 (heterosexual) vs. *n* = 72 (LGBTQ+)] = 15.21, *p* < 0.0001} or disagree {*X*^2^ [1 d.f., *n* = 325 (heterosexual) vs. *n* = 72 (LGBTQ+)] = 5.48, *p* = 0.019} [Figure 12B(v)]. No other statistically significant associations were identified.

#### 3.7.8. “Veterinary students are more vulnerable to discrimination than qualified veterinary surgeons/veterinary professionals”

Most respondents agreed with the statement (177 out of 403) (Figure 13A). However, respondents in the age categories of 24–27 and over 27 years were more likely to strongly disagree than respondents under 24 years {*X*^2^ [2 d.f., *n* = 289 (under 24) vs. *n* = 95 (24–27) vs. *n* = 19 (over 27)] = 7.55, *p* = 0.023} [Figure 13B(i)]. Male respondents were more likely to agree than female respondents {*X*^2^ [1 d.f., *n* = 350 (female) vs. *n* = 51 (male)] = 10.28, *p* = 0.001} [Figure 13B(iv)]. No other statistically significant associations were identified.

#### 3.7.9. “Veterinary students are less likely to report discrimination due to fear of consequences”

Most respondents agreed (204 out of 403) or strongly agreed (128) with the statement. Respondents with a disability were more likely to be strong in their agreement {*X*^2^ [1 d.f., *n* = 368 (no disability) vs. *n* = 35 (disability)] = 9.45, *p* = 0.002} compared to those with no disability being more likely to agree vs strongly agree {*X*^2^ [1 d.f., *n* = 368 (no disability) vs. *n* = 35 (disability)] = 5.65, *p* = 0.017} [Figure 14B(ii)]. Male respondents were more likely to strongly disagree compared to female respondents {*X*^2^ [1 d.f., *n* = 350 (female) vs. *n* = 51 (male)] = 13.79, *p* < 0.0001}, although numbers in the strongly disagree category were low. No other statistically significant associations were identified.

#### 3.7.10. Qualitative responses: awareness and potential solutions

Respondents were given the option to leave any comments or opinions at the end of the survey, which were sub-categorized with qualitative content analysis. Responses were grouped into: “ambivalence/apathy/denial” of diversity and discrimination as an issue in the veterinary profession (Supplementary Table 5) and “consciousness of the importance/concern” about diversity and discrimination in the veterinary profession (Supplementary Table 6). These respondents especially recognized the importance of widening participation in the profession from lower-income families:

“I think with regards to the issue of the lack of racial diversity at vet school you'd to first address the lack of socioeconomic diversity. The majority of people (at least in my year, at my uni) are from wealthy middle class backgrounds. There are a lot of barriers to getting into vet school if you're from a working class family or lower income area.”

“We always talk about the gender unbalance in vet school, but NEVER the racial inequality–while the proportion of ‘black, Asian and minority ethnic’ people in the England and Wales is 14% (2001), number of BAME people within the veterinary profession is 3%. Huge skewed population within vet school, including an over-representation of middle and upper class students compared to lower and working class (race also intersects with this factor too as BAME people make up bigger proportion of the working class now). So while it's important to keep pushing for women's equality, we should definitely not be neglecting pushing for racial, class and LGBT at the same time (if not more), because we're not the only ones who are held back.”

Many respondents, particularly from minority backgrounds, made suggestions on how the profession could improve diversity and decrease discrimination that the authors share (Supplementary Table 7). Some examples of suggestions are supporting colleagues and students and not being bystanders when discriminatory comments or behavior are noticed, profession-wide discussion of the subject, more transparent reporting processes, bottom-up leadership, and minimum standards in practices that take EMS students.

## 4. Discussion

### 4.1. Summary of findings

In the current study, there was a higher proportion of discrimination perceived by the respondents at 36.0% compared to 29.5% and 24% reported in the similarly categorized BVA discrimination and Voices surveys, respectively (19). Most of the discrimination perceived was sexism with racism/xenophobia and ageism being second and third, respectively. Socioeconomic status was commonly referenced within the category of “other” under type of discrimination. Although socioeconomic status is not a protected characteristic under the UK Equality Act 2010 (17), it is a significant intersectional factor to consider alongside race/ethnicity. As a proportion, respondents with one or more protected characteristics were more likely to have experienced or witnessed discriminatory behavior or comments than respondents that did not have a protected characteristic. The type of discrimination experienced or witnessed by individuals with a protected characteristic was more likely to be targeted against that particular protected characteristic.

The described incidents occurred in multiple areas of clinical practice. Most experiences of discrimination were reported in farm animal practice, and most witnessed incidents were reported in small animal practice. The equine practice was third in both experienced and witnessed incidents. The proportions of types of discrimination (ableism, ageism, racism/xenophobia, sexism, and LGBTQphobia) were broadly similar across the professional sectors. The perpetrators were equally likely to be the public or veterinary surgeons. This shows people that students are likely to have the most contact with during clinical EMS. They are also individuals who are likely to have power over students either as an educator or client in an increasingly client-centered communication approach (31). The role of perpetrators had no bearing on the type of discrimination perpetrated.

Reporting of incidents by respondents to this study was much less than that reported in the BVA study at 8% of experienced and 3% of witnessed incidents in this study compared to 19% of students in the BVA study (19). The most common reason selected in this study for not reporting was not thinking anything would be done in response to a report. This perhaps plays less into a power dynamic than other reasons given such as fear of consequences (the third most common reason cited).

### 4.2. Implications

#### 4.2.1. Ageism

This study showed that veterinary students in their clinical years who are over the age of 24 years experienced significantly more ageism as a percentage of total discrimination encountered compared to students under the age of 24 years. This is an interesting result as it has been shown that age preferences in most workforces follow a curvilinear pattern of highest when particularly young or much older (32, 33). One explanation for respondents aged >24 years experiencing and witnessing more general discrimination as well as ageism is that with maturity comes a greater awareness. However, Banerjee demonstrated that there was no significant association between age *per se* and perceptions of discrimination and further postulated that the reason why older students perceived more ageism and other discriminatory behaviors may be that these students had a prior university degree (34). What was shown in the same study by Banerjee that may be an explanation as to why older students perceive more ageism and other discriminatory behaviors could be due to having a university degree (34). Older veterinary students typically have a prior degree when entering vet school, 50% compared with 0.7% under 24 years in the current study's dataset, so may more frequently identify discrimination. This may suggest that once undergraduate veterinary students have completed their degree, they may be better equipped to identify discrimination that they encounter in practice.

#### 4.2.2. Ableism

Ableism as a concept in veterinary medicine has not been studied to date. However, with a growing awareness of ableism in the medical profession against its members (35) and against patients (32) and the fact that 6.7% of respondents in the BVA discrimination survey report having some sort of disability (19), it is important to address this issue. In this study, 8.7% of respondents reported having a disability or chronic illness, half of whom experienced or witnessed discriminatory behavior or comments half of which were categorized as ableism. While many veterinary professionals will be aware of the physically demanding nature of working in clinical practice, it remains important to make reasonable adjustments for those with additional needs (33). Furthermore, removing ableist microaggressions targeted toward students both in education and seeing clinical practice as well as other veterinary professionals can reduce the mental harm to individuals (36). In addition to benefitting students with chronic illness or other disabilities, creating a supportive work environment with sufficient consideration for rest, support, and work–life balance can lead to greater workplace retention, an important issue in today's veterinary profession (37). Ableism in the veterinary profession is also likely borne of the intergroup relations theoretical framework where the in-group perceives the student with disability as somehow being less productive than an abled student and therefore not “pulling their weight”, and so relegated to out-group status (21). Evidence shows that people with non-physical disabilities have more negative experiences in the workplace compared to those with physical disabilities (38). This point is perhaps particularly relevant as most of the disabled respondents to this survey had non-physical and/or invisible disabilities with 11 reporting having dyslexia and seven reporting having some type of disability that caused pain but was invisible, e.g., chronic migraines and rheumatoid arthritis. In addition, it has been suggested that accommodations or differences in performance are seen as less justified by others in the workplace when the disability is invisible (38).

It has been suggested that a strategy for ableism prevention would be better as means of protecting students with disability rather than relying on reaction after the fact (39). Lett et al. showed that disabled university students who experience ableism in the form of microaggressions or overt discrimination have higher levels of anxiety and depression and less confidence in their academic abilities (39). Steps to prevent microaggressions and overt discrimination are important to prevent these negative outcomes. The same study by Lett et al. also shows that support provided by universities to students after they experience ableism does not seem to improve mental health symptoms (39). One such strategy of prevention the authors suggest would be through diversity and inclusion awareness training of EMS placement providers. This training should include examples of ableism and try to break the paradigm that someone with a disability is less productive than other members of the team.

#### 4.2.3. Racism/xenophobia and socioeconomic status

In a study by Mills et al. (40), veterinary students believed that cultural differences did not play an important part in consultations and client communication. This was because the focus of such interactions is always on the patient rather than the humans involved. However, as explained by Mills et al.'s culture, ethnicity, and background can impact effective communication between clients and vets. Discriminatory behavior and prejudicial judgement, therefore, have potential ethical implications for animal welfare. To illustrate this, we highlight a quote from one respondent in the current study who noted that clinicians in practice might alter their clinical plans based on cultural observations:

“I have seen many staff members of different clinics make offhand remarks about the class and race of clients (not to their face) but not act on it. However, a few have commented on how it affects their clinical judgement i.e., not offering certain treatments on the assumption that the client would not want or couldn't afford it without discussing this with the owner.”

Whilst some students expressed abundant consciousness of the issues of diversity and discrimination, others exhibited viewpoints that are consistent with what has been described in a study by Swim et al. as “modern racism”. This is the belief that any inequality which exists is coincidental and due to a lack of merit on the part of minorities rather than systemic injustice (41). However, as evidenced in Supplementary Table 4, students from different white and non-white ethnic backgrounds are having racist and xenophobic encounters that are likely to have an impact.

The BAME respondents who strongly disagreed with the statement “The racial diversity in the veterinary profession needs to be increased” could be concerned about tokenism, i.e., individuals with minority characteristics would be accepted into vet school simply due to those characteristics rather than for their abilities. As highlighted in the qualitative responses when sharing opinions showing ambivalence or denial with regard to diversity (Supplementary Table 5), and the example shown. It is worth highlighting that racist incidences were likely related to the context in which data were collected (March and April 2020) as negative feelings and discrimination against Chinese people (and those who were thought to be Chinese) were increasing across the world at this time (42). This plays into the perception of threat from someone not part of the usual group. A personal reflection by Gao and Sai also noted more anti-Chinese racism in the UK during the coronavirus pandemic (43), suggesting that COVID-19 was an excuse for airing racist opinions rather than fears of a virus such as SARS and COVID-19. Indeed perceived public health threats from majority Caucasian countries (such as BSE from the UK or swine flu from the United States) did not result in such racial backlash compared with majority BAME countries or continents (such as SARS and COVID-19 from China or Ebola from Africa) (43).

Initiatives to improve racial diversity in the veterinary profession exist at present in an attempt to counter racial inequalities. One idea is to encourage more applications to vet schools from underrepresented groups but maintain the same selection criteria to try and mitigate any perceptions of competition between groups. This would help to address potential unconscious bias or overt opinions on this subject and to alleviate those concerns that people may have.

Some respondents also made the argument that we should not show deference to potential students on the grounds of ethnicity but instead socioeconomic status to increase diversity in the profession. Potential concerns about tokenism could be further explained by the research by Dover et al. which lists the possible unintended negative consequences of initiatives to increase diversity (44). For example, when those in charge of diversity initiatives intend to send the message that “underrepresented groups are treated fairly”, it can have potential unintended consequences such as people underestimating anti-minority discrimination, overlooking/dismissing/delegitimising discrimination claims, and derogating minority discrimination claimants (44). In addition, perceptions that marginalized identities are positively selected for can result in a belief that individuals from marginalized groups are therefore undeserving of their place, of lower ability, and “taking the place” of someone deemed to be more deserving. The “resource” of a vet school place or an EMS placement is seen as scarce and the marginalized student is placed in the out-group as a competitor (21). It is, however, the case that BAME people are more likely to be of lower socioeconomic status (45). It is likely that it is the intersection of multiple factors including race and socioeconomic status as well as many more factors at play in different proportions within each unique respondent. The authors suggest that research using an intersectional framework approach, as discussed by Gayles and Smith (46), looking into the effects of ethnicity and social mobility on students' access to and experiences in the veterinary profession, is critical.

Class or socioeconomic background is not a protected characteristic in the Equality Act 2010 and therefore, the effect of class on encountering discrimination was not directly investigated in this survey. However, of those students in our survey who chose the “Other” characteristics that discrimination was witnessed against, class or socioeconomic background was the most common characteristic described. This finding may be particularly relevant to the veterinary profession as we know that 33.8% of vets attended private schools (47) compared to 7% of the UK population (48) which suggests a bias in veterinary medicine for those from families of greater economic means.

#### 4.2.4. Sexism

Many examples of sexist discrimination were found in this study, of which the common sub-categories can be seen in Supplementary Table 5. Sexism was the most common form of discrimination encountered, which is consistent with the findings from the BVA discrimination study (19). This raises questions as to whether the problems the profession has with the retention of vets (49), particularly around retaining women vets (47), lower levels of ambition in female vs. male veterinary students (50), and altered career aspiration (51) could be related to the discrimination, doubting of their abilities, and stereotypic assumptions that female vets experience from their student days onwards. This echoes the idea that the profession is feminized but not feminist and still functions in a male-dominated manner (18).

Recent research by Wayne et al. shows that 73% of American veterinarians who are mothers said that they had perceived discrimination due to their maternal status (52). Such content was also evident in qualitative responses in the current study. For example, comments encouraging young female vets not to have children. Frequent examples of sexism echo those related to ableism. For example, female vets are likely to leave to have children, are therefore less productive, and not “pulling their own weight” compared with other members of the team. It is again likely that the picture is more complex with an intersection of gender and disability status since most respondents with a disability were female.

The authors argue that this research on discrimination has the potential to further inform other related issues the veterinary profession has, for example, with retention and the burnout of female vets (53). If the veterinary profession wants to ensure the best young minds in veterinary medicine reach their full potential, this issue must be addressed with urgency. This research shows (Figure 6) that when it comes to discrimination and equality, some male veterinary students are ambivalent, apathetic, or deny issues of diversity and discrimination in the veterinary profession. The authors suggest that this means that more work needs to be done to improve the culture and beliefs held across the generations in veterinary medicine, which contributes to inequalities such as men still outnumbering women in senior veterinary roles (49).

#### 4.2.5. Homophobia/LGBTQphobia

The findings suggest that veterinary students could benefit from more awareness about what their colleagues are experiencing in terms of discrimination based on their sexuality. Approximately 51.4% of the current study's respondents who identified as LGBTQ+ said that they had experienced or witnessed discrimination of some kind during their clinical EMS placements compared to 33.9% of heterosexual respondents. The Veterinary Voices Survey Spring 2021 found that 24% of LGBTQ+ respondents experienced or witnessed discrimination compared to 14% of heterosexual respondents (28). Not all LGBTQ people are necessarily “out” at their clinical EMS placements which may explain why they did not necessarily experience discrimination. The fact that not all students feel comfortable sharing information about themselves could be related to the fact that they are only at EMS placements for a short time and/or are less familiar with the staff. In explanations as to why incidents were not reported, wanting to avoid controversy on their placement and risk ostracism was highlighted (Supplementary Table 2).

The findings indicate a lack of communication about the processes of reporting discrimination within veterinary businesses. Respondents were often unsure or unaware if reports were made and how they were dealt with. It is possible that it is not always appropriate to inform all parties about the outcomes of a report, but greater transparency about the procedures appears to be needed whilst managing the challenges of navigating such reports.

The authors suggest EMS students are more vulnerable in terms of being discriminated against as they experience some unique pressures and therefore feel less able to report discrimination (Supplementary Table 2). Indeed, high levels of discrimination and low levels of reporting have also been found amongst UK medical students, and they echo similar reasoning behind this to the veterinary students in the current study (20). Improving the culture of workplaces to have a no-blame ethos (such as in strategies to improve reporting of medical errors) has been demonstrated to improve reporting (54). This may be beneficial in improving matters in veterinary workplaces. Respondents stated that they would put up with discriminatory behavior or comments to not have to organize additional placements. They also cited the fear of a “bad review” resulting in the expectation to repeat a placement as another reason. This reinforces the power dynamic paradigm that is pertinent to students on placement. The authors expect this problem to only increase with greater competition for EMS placements going forward. It is worth noting, however, that in the current study of the few respondents who did report to have personally experienced discrimination, only two were not satisfied with the response (Figure 5C). This differs from the experiences reported in the BVA Discrimination Survey. In the BVA survey, 71% (19) and 56% in the Spring 2021 Voices Survey (28) were not satisfied with the outcome after reporting the incidence, although numbers reporting were very low. This could suggest that university reporting processes, when utilized, do give those reporting a more satisfactory response than the processes employed in the general veterinary workplace.

Since the collection of the data in the current study, special interest groups organized by minorities within the UK profession [such as the British Veterinary Ethnicity and Diversity Society (BVEDS), the British Veterinary Chronic Illness Support (BVCIS), and the British Veterinary LGBT+ Society (BVLGBT+)]have had the space to start leading conversations to begin a process of change. Larger veterinary bodies such as the BVA have responded to these conversations with campaigns such as the Good Workplace Guide and a microaggressions poster campaign. It would be of interest to repeat this study in the near future to measure the impact of these initiatives.

### 4.3. Limitations

The authors acknowledge that there is a relatively small sample size for the study. The majority of participants were women (86.8%) which reflects the demographics of veterinary students. This is especially relevant considering the specific demographic groups that were used for the analysis. For example, due to the small number of students in our respondent population in each of the groups of Black, Asian, and other minority ethnic students, these categories were grouped for analysis as “BAME” respondents. The same is also true for LGBTQ+ respondents. Likewise, the same was true for the wide variety of visible, invisible, physical, and mental disabilities that veterinarians and veterinary students may have which have been grouped in this study. It is important to remember that these groups are not homogenous and likely encompass unique experiences and differences. More studies with larger sample sizes are needed to further determine the nuanced experiences of discrimination within these groups, and further qualitative research would also assist in this endeavor.

In addition, it should be noted that this was a study based on voluntary participation. Evidence shows those with greater interest in a survey topic state that they are more likely to respond to it (55). It is reasonable to expect that those who have encountered discrimination would have increased investment in the issue. Bearing this in mind, the proportion of those that report experiencing discrimination in the current study may be higher than the true figures (54, 55). Therefore, this study cannot gauge the prevalence of discrimination experienced or witnessed by students in clinical EMS. However, the study can be used to inform the general picture of what discrimination experienced or witnessed looks like, where it happens, who by, and student opinions on it. Thus, the veterinary profession can use this information to begin to take positive action.

Looking into discrimination that vet students experience in other areas of their education was beyond the scope of this study. Nevertheless, several respondents commented that they wished to be able to share their experiences of discrimination whilst undertaking Animal Husbandry Extra-Mural Studies (AHEMS) or pre-clinical Extra-Mural Studies.

Intersectionality as a theoretical framework has been considered in terms of age, disability status, race/ethnicity, gender, and LGBTQ+ status. Use of intersectionality could not be exhaustive for all potential characteristics and their interrelations with one another, but its use is one of the key strengths of the research. The authors hope these findings will enable further research, discussion, and consideration of intersecting factors in the study of discrimination in the veterinary profession.

### 4.4. Conclusion

Discrimination is a significant issue affecting veterinary students in the context of EMS. The discrimination experienced and witnessed by veterinary students in this study is broadly similar in character and distribution to the discrimination reported in the BVA's report examining the wider profession. Students are in a particular position of vulnerability in terms of the power dynamic between them and perpetrators of discriminatory behavior. Students with either one or more protected characteristics experience and/or witness more incidents targeted toward their characteristic(s). This study raises questions as to how some veterinary professionals are conducting themselves at work in terms of professionalism and inclusivity, and their reinforcement either knowingly or unknowingly of a hierarchical in-group/out-group relationship with students.

A cultural and institutional shift is needed to combat discrimination within the veterinary profession and to recognize the impact of certain behaviors. It is important to better support staff and students who encounter such behavior particularly when the client-centered communication focus can place clients in perceived positions of power. It may be interpreted that failure to recognize discrimination is a generational issue, with older generations being less likely to be aware of discrimination. However, the data presented in the current study show that this discriminatory attitude persists in some current students, and therefore, any educational strategies addressing the subject of diversity and inclusion should cover the entirety of the profession including current students.

Members of minority groups are the people who experience and witness the most discriminatory behavior. Our findings suggest that when there is an intersection of more than one marginalized identity, discrimination can be amplified and we highlight this as an area for future research. It is important that those with protected characteristics must be listened to and included in discussions and strategies to improve diversity and the working experiences of all members of the veterinary profession. This is key to avoiding tokenism and recognizing and defining the microaggressions that contribute to a climate of hostility.

Finally, to fully realize the problem of discrimination within the veterinary profession and evaluate the steps taken to address the problem, reporting processes need to be transparent, safe, and well-communicated.

## Data availability statement

The raw data supporting the conclusions of this article will be made available by the authors, without undue reservation.

## Ethics statement

The studies involving human participants were reviewed and approved by University of Surrey Ethics Committee. Written informed consent for participation was not required for this study in accordance with the national legislation and the institutional requirements.

## Author contributions

CM contributed to the conception and design of the study, performed the statistical analysis, and created the figures. OS and RM developed and tested the questionnaire and gathered data. KH provided a second opinion on the qualitative content analysis. OS wrote the first draft of the manuscript and created the supplementary tables. CM and KH revised and edited the manuscript. All authors contributed to the article and approved the submitted version.
