# Supplementary material for: A cross-sectional study examining perceptions of discriminatory behaviors experienced and witnessed by veterinary students undertaking clinical extra-mural studies
Source: Front Vet Sci. 2023 Apr 28;10:940836. doi: 10.3389/fvets.2023.940836 (PMC10175701; doi:10.3389/fvets.2023.940836)
Supplement: Supplementary file 1 [file Data_Sheet_1.docx]

Supplementary Material

# Questionnaire

1. Which vet school do you attend?

- Surrey
- Bristol
- Cambridge
- Dublin
- Edinburgh
- Glasgow
- Nottingham
- Liverpool
- RVC

1. Which year are you in at vet school? (If you’re not 3rd year or above this questionnaire does not apply to you)

- 3rd year
- 4th year
- 5th year
- 6th year (Cambridge)

1. What level of education were you when you entered your Veterinary Medicine degree?

- Postgraduate
- Undergraduate

1. How old are you?

- 20 - 21
- 22 - 23
- 24 - 27
- 28 - 35
- 36 - 45
- 46+

1. What is your gender?

- Female
- Male
- Other (please specify)
- Prefer not to say

1. Was this your assigned gender at birth?

- Yes
- No
- Prefer not to say

1. What is your ethnicity?

- White English / Welsh / Scottish / Northern Irish / British
- Gypsy or Irish Traveller
- Irish
- Other white background (please specify) [TEXTBOX]
- Black Caribbean
- Black African
- Other black background (please specify) [TEXTBOX]
- Other mixed/multiple background (please specify) [TEXTBOX]
- Indian
- Pakistani
- Bangladeshi
- Chinese
- Other Asian background (please specify) [TEXTBOX]
- Arab
- Other (please specify) [TEXTBOX]
- Prefer not to say

1. What is your sexual orientation?

- Heterosexual
- Homosexual
- Bisexual
- Queer
- Other (please specify) [TEXTBOX]
- Prefer not to say

1. Have you ever **experienced** discrimination whilst undertaking your clinical EMS placements?

- Yes
- No
- Not sure

1. Do you have a disability?

- Yes (please specify) [TEXTBOX]
- No
- Prefer not to say

**If answered yes/not sure to experiencing discrimination:**

1. How many times have you experienced discrimination in veterinary practice?

- 1
- 2-3
- 4-6
- 7-9
- 10+

1. When did the discrimination occur? (please select all that apply)

- 2nd year summer (Surrey)
- 3rd year
- 4th year
- 5th year
- 6th year

1. In what type of practice(s) did it occur? (please select all that apply)

- Small animal
- Farm
- Equine
- Exotics/NTCAs
- Mixed (please specify) [TEXTBOX]
- Other (please specify) [TEXTBOX]

1. Which characteristic(s) were discriminated against? (please select all that apply)
   - Age
   - Disability
   - Gender identity
   - Marriage or civil partnership
   - Pregnancy and maternity
   - Race
   - Religion or belief
   - Sex
   - Sexual orientation
   - Other (please specify) [TEXTBOX]
2. Who discriminated against you? (please select all that apply)

- Vet
- Vet nurse (RVN)
- Another student (nursing or vet)
- Member of the public
- Reception/administrative staff
- Veterinary care assistant (VCA)
- Other (please specify) [TEXTBOX]

1. Please give a brief description of the incident and the impact it had on you, if multiple please describe each separately

[TEXTBOX]

1. Was the discrimination reported? (please select all that apply)

- Yes
- No
- Not sure
- Prefer not to say

1. If you **did** report it, were you satisfied with the response? Please give more details if you wish

- Yes
- No
- Not sure
- [TEXTBOX]

1. If you **didn’t** report it, then why not? (please select all that apply)

- Concerned about the consequences of reporting
- Didn’t want other people to find out about the incident
- Didn’t know how to report
- Don’t see why I should report it
- Didn’t think there would be anything done about it
- Someone else reported it (please specify) [TEXTBOX]
- Other (please specify) [TEXTBOX]

1. Have you ever **witnessed** discrimination against another individual/group whilst undertaking your clinical EMS placements?

- Yes
- No
- Not sure

**If answered yes to witnessing discrimination:**

1. How many times have you witnessed discrimination against veterinary students when undertaking clinical EMS placements?

- 1
- 2-3
- 4-6
- 7-9
- 10+

1. When did this occur? (please select all that apply)

- 2nd year summer (Surrey)
- 3rd year
- 4th year
- 5th year
- 6th year

1. In what type of practice(s) did it occur? (please select all that apply)

- Small animal
- Farm
- Equine
- Exotics/NTCAs
- Mixed (please specify) [TEXTBOX]
- Other (please specify) [TEXTBOX]

1. Which characteristic(s) were discriminated against? (please select all that apply)

- Age
- Disability
- Gender identity
- Marriage or civil partnership
- Pregnancy and maternity
- Race
- Religion or belief
- Sex/gender
- Sexual orientation
- Other (please specify) [TEXTBOX]

1. Who did you witness being discriminatory? (please select all that apply)

- Vet
- Vet nurse (RVN)
- Another student (nursing or vet)
- Member of the public
- Reception/administrative staff
- Veterinary care assistant (VCA)
- Other (please specify) [TEXTBOX]

1. Please give a brief description of the incident and the impact it had on you, if multiple please describe each separately

[TEXTBOX]

1. Was the discrimination reported? (please select all that apply)

- Yes
- No
- Not sure
- Prefer not to say

1. If you didn’t report it, why not? (please select all that apply)

- Concerned about the consequences of reporting
- Didn’t want other people to find out about the incident
- Didn’t know how to report/who to
- Didn’t see why I should report it
- I didn’t think it would help the situation
- Someone else reported it (please specify) [TEXTBOX]
- Other (please specify) [TEXTBOX]

**All respondents should answer this question:**

Please rate your opinions about discrimination in the veterinary profession: (tick one box per statement)

|  | Strongly Agree | Agree | Neutral | Disagree | Strongly disagree |
| --- | --- | --- | --- | --- | --- |
| Discrimination against veterinary students is an important issue |  |  |  |  |  |
| Discrimination against veterinary students happens frequently |  |  |  |  |  |
| Discrimination against female vet students is no longer an issue due to them being the majority |  |  |  |  |  |
| Older students are more respected so are discriminated against less |  |  |  |  |  |
| The racial diversity in the veterinary profession needs to be increased |  |  |  |  |  |
| Known LGBTQ+ status causes an increased risk of discrimination for vet students |  |  |  |  |  |
| Vet students are more vulnerable to  discrimination than qualified vets/veterinary professionals |  |  |  |  |  |
| Vet students are less likely to report discrimination due to fear of consequences |  |  |  |  |  |
| Governing bodies e.g. BVA/AVS/RCVS/Vet schools etc. are doing enough to tackle discrimination |  |  |  |  |  |

1. If you have any other opinions not included above regarding discrimination against veterinary students please write them below…

[TEXTBOX]

# Supplementary Tables

**Table 1** Incidents of discrimination against class/socioeconomic background witnessed by respondents

| ‘Other’ characteristic reported | Description of discrimination witnessed |
| --- | --- |
| ‘Socioeconomic [status]  Having an accent’ | ‘Not treated with same respect  Dismissive’ |
| Class | ‘Working at a **charity hospital**, **comments about the intelligence of clients on benefits were common from the vets and vet nurses**. This didn't sit right with me because it's very prejudice and not the thinking a medical professional should have towards the clients they are there to help. …’ |
| Social class/background  Perceived intelligence | ‘Frequently at several of my placements (**especially at charity run practices**) there would be offhand comments about some of the clients and sometimes some rather disrespectful views aired never to the person in question but in open conversation with other staff members’ |
| Race, Class | ‘I have seen many staff members of different clinics make off hand **remarks about the class and race of clients** (not to their face) but not act on it. However a few have commented on how it **affects their clinical judgement** I.e. not offering certain treatments on the **assumption that the client would not want or couldn't afford it** without discussing this with the owner’ |

**Table 2** Common codes in descriptions of sexism against women experienced or witnessed by respondents, with examples

| Common Code | Frequency | Examples |
| --- | --- | --- |
| Comments about females not being as capable vets (particularly seen in farm practice) | 31 | ‘**Older male vet told me that I should be a [housewife] rather than a vet** and said 'you need to be strong to be a farm vet' implying I couldn't because I'm a shorter woman. He also told me **women should obey men’**  ‘A client made a sexist comment to me about my low ability to be a large animal vet since I am a girl and **girls don't like hard work but would rather stay at home and put makeup on**.’  ‘Repeated **invalidating comments about capabilities with horses as a female**.’  ‘The owner and head vet in a large equine referral centre was incredibly rude to myself and another female vet student who went at another date and said, and I quote, **‘women can’t be as good vets as men can’**’  ‘On a farm outing a **vet and a farmer were discussing the fact that they couldnt imagine i would be able to do an LDA surgery due to the fact that I was young and female** and therefore weak and inexperienced.’  ‘..General comments about how veterinary work must be difficult for women, lack of strength etc…’  ‘…I have had people tell me that women shouldn't be large animal vets because we are weaker, smaller, and (quite memorably), one lady said  **"one kick in the wrong place and that's it, a lady can't have children anymore"…**’  ‘comments from the farmer on being a "small, weak female"…’  ‘On farm placement I was **told by the farmer that I was at the wrong type of vet practice and was asked why I wasn’t ‘playing with cats and dogs’**  **instead** which I would say was sexual discrimination as he was insinuating that I wouldn’t be strong enough to handle larger animals.’  ‘Multiple sexist comments at many farms visited is quite common (seen practice mainly in Somerset). Mainly **underestimating my strength, ability** to handle cattle, or intention to enter farm medicine…’  ‘Castrating calves - a farmer commented that it was **‘a bit brutal for a girl’** and i said it was a good learning opportunity and he said something about being a lesbian…’  ‘Farmer making those sadly **'normal' comments** about me looking too young and **being female so not physically able to do a good job**. Asking the vet I was with **why they let girls do this sort of work**. Also I was very disappointed that the vet I was with (a younger male vet) did nothing to counter this view but just laughed along leaving me feeling very inadequate and hugely doubtful of my own abilities’  ‘**I wasn't strong enough or able to do something because I'm a woman**. In general working with cattle. There was a farm call where the client was surprised that there was a woman who had an interest (and ability) to work with cattle…’  ‘Sexist incidents-usually when dealing with large animals (farm animals and horses) **owners/farmers think I am incompetent just because I am a woman of small stature, they think I lack a man’s physical strength**.  It is disheartening, I totally gave up on doing farm animal work because I cannot deal with the farmer’s attitude…’  ‘…With regards to my gender it is more to do with strength and which tasks i can complete because “i am a girl”. Such as “oh don’t carry this, it’s really heavy, **let your “male colleague” carry it” or “be careful with this horse, wouldn’t want to break your delicate body”.**’  ‘The incidents I have encountered have all been while on farm calls. I have just had a few side comments from farmers - **'I'm sure you want to do small animals - it requires less strength'** or **'another bloody female vet.'** I think there is still a stigma among certain farmers that females are not capable enough to handle large animal work. I would like to enter farm animal practice so I find it frustrating to see this mentality is still present. **It makes me feel like I have to try twice as hard to make an impression once I am in practice.’**  ‘Gender discrimination, specifically that I’m female interested in farm practice and **presumed to be of poorer quality than my male counterparts**’  ‘…**Once a farmer told me I couldn't do the job of a male vet as women aren't strong enough**. This resonated with me, to the point where I am going into small animal practice when I graduate.’  ‘…lots of backhanded stories about female vets being 'slower' than the male ones…’  ‘The main situation I recall involved a **male farmer telling me I wouldn't be strong enough to carry out a certain job and that his son would be better**.’  ‘Farm: being underestimated due to being a female and **asking for confirmation of diagnosis from male student next to me**. Blatant misogyny from farmers and other workers…’  ‘Not sure if it’s exactly discrimination but found a lot of **farmers pointing out you’re a girl and might not be able/strong enough to do some of the work a farm vet has to do**. I think this is quite common for a lot of girls wanting to go into this field…’  ‘**Male farm vet told me I wouldn't make a good farm or mixed vet because I was a woman** it meant I was a) less able to do manual tasks, and b) would want to have babies which wouldn't suit on call because I couldn't leave a kid home alone…’  ‘Vet was inappropriate when talking to me and consistently **talked about how women weren't cut out to be farm vets**.’  ‘The vet at the practice seemed to undervalue me and didn’t let me do a lot. He would constantly make **remarks about me not being up to standard or not being good enough.** Despite being in second year with no clinical teaching, it being my first clinical placement and having no opportunities to show him what I could do. **I felt as though this was due to my sex**. He was the only vet in the practice and all the other staff were female nurses or female work experience. **I got the impression he gave himself the position of being the man in charge**.’  ‘**On Farm visits with a young female vet some farmers would be less cooperative or make [unnecessary] comments** about the fact that the vet was young and female suggesting that that might affect the quality of their work.’  ‘…Young vets and females discriminated against by clients wanting the older male vets because they think their care is better (even when similarly qualified)…’  ‘**The specialized opinion of a woman with more years [of] experience and board certification was ignored for that of a man not even graduated 5 years. All the men in the room deferred to him**. She's new to the region, but has done good veterinary work there, but her qualifications didn't seem to matter at all.  Another time we were on a farm call, and a farm worker wouldn't speak to her directly, just kept telling his boss that what she said happened couldn't have possibly happened.’  ‘…**Female small animal vets get questioned on whether they know as much as their male colleagues**. These frustrated every female vet I worked with…’  ‘Usually on farm with female vets in a very traditional area. **Farmers doubt ability of female vets** and one even rang the practice to complain that they had sent a girl and not a male vet.’  ‘…Client told the female vet tech to get a male vet tech to hold her dog because they didn’t believe that they were strong enough…’ |
| Female students being prevented from doing things on clinical EMS/male students being favoured for clinical tasks | 21 | ‘…I was not allowed to restrain the horse because we **needed a big strong man** to hold him.’  ‘Small animal RVN (female) was consistently rude to me and **blocked my advances to further my skills**, …. She did not do this with male students, and **self-proclaimed that she preferred male students**.’  ‘[female vet] asked me to scrub in to assist [with c-section]. The older male vet turned up, **told the farmers son to scrub in** with him and proceeded to order him to act as the assistant holding the uterus, **with myself renegated to instrument holder**. … It seems like such a trivial thing but **it had a profound affect on me and my attitude to large animal studies for months afterwards**.’  ‘Farm- **wouldn't let me do any clinical skills** thinking I would be incapable as a female for anything slightly heavy’  ‘Went to a farm on a TB testing call and had the whole day having the farmer, his workers and the vet joking about me being a woman. **Was given no hands on experience in case I hurt myself** or if the animals hurt me’  ‘**Generally ignored when a male vet student (similar age, year group, and experience with horses) joined me for my 2nd week of equine preclinical. He was favoured to do any clinical task**, and the attitude of the male vet who I had spent time with in the previous week changed dramatically; I was suddenly ignored. Not sure this was discrimination, and it definitely wasn't serious, but it could have affected my learning.’  ‘**Primarily a male vet making comments about females being incapable on farm and refusing to take students out**. Also a different vet commenting on how a particular male vet only took the male or very attractive female students out with him.’  ‘Many comments made about being a woman, **tasks given to male colleague despite him showing incompetence**, more forgiving to male colleague.’  ‘Inappropriate jokes about me being female and refusing to let me do things because of it’  ‘**Was prevented from taking part in a C-section surgery and medical treatment of a batch of calves due to the fact I was a female vet student**  Farmer decided that male students had a shorter vet programme in college so I was underqualified compared to the male students he had seen before on his farm.’  ‘Myself and another vet student were on placement the same week. **There was a claw amputation to be carried out on a cow in the afternoon**. One of the senior partners asked the other student, who was male, to come with him as he might **"need some man power"**. I had been with another vet TB testing in the morning and **this meant I also went TB testing again in the afternoon; we had been meant to switch over**.’  ‘…I was on placement with a 4th year male vet student (I am female) from the same university, and **both the male vets let the 4th year student do all the surgeries while I (the 5th year) had to watch**…’  ‘…Farm and equine owners have also **asked that I keep back from their animals on visits and wait in the male vets cars because I was a young girl** and they didn’t want me getting hurt.’  ‘**Two of us (female students) were barely given any case load/involvement** in tasks (eg surgeries) but the **Male student** (in the same year, same class as us) were **given more opportunities for involvement**’  ‘..I have been **told by a vet that as a woman** (Btw I grew up on a farm, and want to be a farm vet) **I should stand back and watch as they were doing "dangerous" procedures** (castrating and dehorning)…’  ‘…Also whilst on placement a few times **I have been ignored in place of a male vet student or they have been prioritised over me**. Very frustrating.’  ‘…There is always a **preference of men being allowed to do things over women** being allowed to do things especially concerning what we are allowed to do on some placements/rotations (mostly small animal)…’  ‘Head male Equine veterinarian … would allow the male vet student to participate and I would have to stand and watch…’  ‘I have experienced while in large animal practice **male students being blatantly favoured over myself** by male partners simply for their gender…’  ‘Someone unwilling to let me observe anything with them and told me I would just be in the way.’  ‘**Nurses being keener to work with male vet student** and bitching about another female vet student. **All nurses were flirting with male student’** |
| Clients having a preference for male vets | 18 | ‘A farmer … said **"all these women wanting to be vets"** in an annoyed tone and tutted, as if he doesn't believe women should be vets. The male vet in the room said nothing.’  ‘I spent some time on a farm being told that “**all these girls” that keep graduating** is too many. And the men are better vets and why are there so many women. He’d only have male vets on the farm and **when we turned up (myself and a female vet) he was furious.**’  ‘**Member of the public addressing me [a male student] as the vet and ignoring the female vet**’  ‘…farmer asked if the new vet would be male because he had had trouble with a female vet before’  ‘… I haven’t experienced many but **few small animal clients said they think a male vet student would be more intelligent and better at doing everything vet related**. Again this is disheartening too and it’s exhausting having to prove yourself every single time whereas your male colleagues are never questioned and they don’t need to put that much effort in building trust with the owners.’  ‘**Male students are always regarded as more qualified/important by the general public**. Whereas I've been [asked] many times by clients what year of school I'm in, if I want to be a vet when I finish school etc even when in my final year, or what age I am which I don’t imagine male students experience as much.’  ‘The incidents I have encountered have all been while on farm calls. I have just had a few side comments from farmers - **'I'm sure you want to do small animals - it requires less strength'** or **'another bloody female vet.'**…’  ‘…Members of the public when I go into consults often say things about how young I am that I can't be a vet student, or that **they prefer "the kinds of vets like *him***, you know what I mean, but times are changing"’  ‘Farm - was told I would make a good vet but **they would pick a man because that's what clients want**…’  ‘A client at the practice would only want older, more experienced men coming to see his herd of cows and **would make a fuss if the vet who showed up was female or young**’  ‘On Farm visits with a young female vet **some farmers would be less cooperative or make [unnecessary] comments about the fact that the vet was young and female**’  ‘… clients not wanting to see female or foreign vets.’  ‘…Young vets and females discriminated against by **clients wanting the older male vets** because they think their care is better (even when similarly qualified)…’  ‘Repeated [member] of the public **trusting myself over the qualified female veterinarian** because they’re a) female, b) look too young to know what they’re doing and c) they just prefer the word of a “bloke”’  ‘Sexism - usually female vets being discriminated. Farm vets request male vets or call the practice and say they don’t want to pay for a female vet (similar incidents with equine but less so)…’  ‘Overheard a conversation between a farmer and male vet. He made a comment **"If you ever quit I will switch practices I can't be dealing with more female vets"**’  ‘Member of the public commenting on **‘lady vets’’**  ‘Usually on farm with female vets in a very traditional area. Farmers doubt ability of female vets and one even **rang the practice to complain that they had sent a girl and not a male vet**.’ |
| Inappropriate sexual remarks/behaviour towards female vet students / blatant misogyny | 10 | ‘**Rude, inappropriate comments** about my gender and **how many men I am ‘probably’ sleeping with**…’  ‘…**Inappropriate comments about my body** / being a young female – made me feel insecure and very uncomfortable’  ‘**I was asked to cook a farmer supper**, and told that **my fiancée had better make me wear my ring** (against dress code) or I’d consider other men’s offers.’  ‘.. Trotted horse up and vet said [they] always get the girls to trot up’ **implying watching them** …’  ‘Head male Equine veterinarian … **pretending to whip me on the bottom with a horse whip and touching my [waist],** … Made me feel very comfortable…’  ‘**Vet told a family member that farmers were sexually attracted to me** and insinuated they were enjoying having me there because of that. It made me feel uncomfortable going back to the practice to finish my week there…’  ‘**Sexual comments made towards myself** by nursing staff and members of the public…’  ‘**Flirting, asked for my mobile number** whilst working with the client, put in **an** **uncomfortable position**.’  ‘Comments made in relation to restraining a sheep for epidural referring to me being female - was very minor but made me feel uncomfortable as both the farmer and vet were male’  ‘Farming clients making **inappropriate sexual remarks** to a fellow vet student  Pet owners making inappropriate sexual remarks to a fellow vet student’ |
| Assumptions/comments about female vets having children | 8 | ‘**Personal questioning about whether I ever had plans to have children**, followed by advice not to and how **inconvenient it was for an employer**…’  ‘… told **women are better suited to small animal side as it's easier to have kids there**, and I should consider that aspect…’  ‘…Small - after CEMS, was told I had done well and **would make a good vet until I decide to go off and have kids**…’  ‘..Saying how I was the **‘wrong sex’ to be a LA vet** because I would [need] maternity leave and I can’t have too many kids - assuming I could have them or want them?...’  ‘…I have had vets complain at me about how there are too many women and the profession is pyramiding because we all go off to have children…’  ‘… Have been told several times that one day I will want children because  **‘all women want children even if they say they don’t’**.’  ‘Was told that my age and generation of vets don't work hard and that we wouldn't be cut out to do the 'real' work that his generation of vets have done. He also said that **because I'll be having children that I will have to work even less**.’  ‘**Male farm vet told me I wouldn't make a good farm or mixed vet because I was a woman** it meant I was a) less able to do manual tasks, and b) **would want to have babies which wouldn't suit on call because I couldn't leave a kid home alone**…’ |
| Preferences for hiring male vets over females | 4 | ‘…**told that** **they would actively overlook me for a job** not because they wouldn’t want me, but **because I’m female** and they can’t afford maternity, so would prefer to hire a male…’  ‘Farm - was **told I would make a good vet but they would pick a man** because that's what clients want…’  ‘Practice was made up of four (male) vets. Told … that one of the partners **refused to hire women vets** or non-white vets.’  ‘… I asked how long it had taken for them to recruit a new vet after one of their colleagues had left. The male vet said **"it was a bloody nightmare trying to replace him- all the people applying for the job were female or foreign".…** women only stay for a year then go off to have babies". I said "not all women want to have babies, and shouldn't we be encouraging men to stay at home to raise children as well?". He laughed and said **" you'll realise when you have children of your own, which I'm sure you will,** that men just biologically [aren’t] suited to it.’ |

Table 3 Common codes in descriptions of student’s encounters with racism and xenophobia

| Common code | Frequency | Examples |
| --- | --- | --- |
| Clients being racist/xenophobic towards vets/students | 13 | ‘A trainer at a yard commented on a previous student who was on EMS before me **saying when he first saw her he thought that she was delivering a takeaway**.’  ‘….Xenophobia- both small and large animal owners/farmers, usually the farmers. **They question if I can understand the concepts well enough or diagnose and treat as well as a British vet student**. I feel like it will be a huge barrier if I wanted to work here and discourages me to stay here after graduation. **I’m thinking of practicing at other English speaking countries after graduation**.’  ‘Assumed that I couldn’t be of veterinary importance because I’m black’  ‘The incidents were mainly related to Brexit being a hot topic and me being European. Some clients make comments when they learn that I am European such as “woaw she is not from this country and can speak perfect english” or **“it’s nothing against you personally, but i just don’t really like foreigners in my home country”**.’  ‘Farmers **treating me like I know nothing/am untrustworthy because I have an American accent**.  Farmers not trusting vets if they are **not** **from Ireland/have an accent**…’  ‘…clients not wanting to see female or foreign vets.’  ‘…**European vets discriminated against by clients** (occasionally because of communication issues but **often with a discriminatory undertone/inappropriate language**)…’  ‘At blue cross, client refused to see either of my Asian colleagues and would only talk to me’  ‘A client used the word “nigger” in a consult’  ‘Farmers not trusting vets if they are not from Ireland/have an accent.’  ‘…Racial "jokes" by clients’  ‘…**several clients discriminating against a fellow student who is Malaysian. Would reply to me instead of her when she asked a question and was leading a consult** (even when I had my back turned/ignored) and one elderly client made uneducated comment asking the student if she knew a random Chinese lady who she knew of...’  ‘Mostly **clients on seeing a Spanish vet began doing the whole talking louder and repeating the same thing over** and over cos they think she couldnt understand them when she was trying to take a history…’ |
| Racism/xenophobia against Asians | 11 | ‘Farmer stated that he was **glad I wasn't a chinese student** because they aren't as smart about how to be around cows.’  ‘….**Questioning from a member of the public about my ethnicity**, an uncomfortable conversation and definitely a case of "yellow fever"  **Disparaging comments about Chinese/Asian nationals** from a member of staff following coronavirus outbreak...’  ‘They were making racist comments about **Chinese people** spreading coronavirus and **being 'disgusting'’**  **‘**verbal discrimination’ – experienced by Chinese student  ‘…There have been **multiple comments** from the same student at vet school **about the Chinese government and how its 'my people doing x' (despite being a British citizen)**. I did find it a bit aggravating as my nationality is not Chinese and **its got nothing much to do with me what the Chinese government is doing**…’  ‘…**I’m Chinese and maybe the farmer feels like I can’t speak English or that I can’t speak well**. And he avoided to acknowledge me when we met and even after hearing me speak in English, **he decided to speak to me through other people, even though I was standing right next to him**.’  ‘Placement would not take student due to asian ancestry’  ‘…Made very racist comments about Asian people.’  ‘…i did placement with my friend who is asian and he reported back to me multiple incidents of farmer discrimination’  ‘…several **clients discriminating against a fellow student who is Malaysian. Would reply to me instead of her when she asked a question and was leading a consult** (even when I had my back turned/ignored) and one elderly client made uneducated comment asking the student if she knew a random Chinese lady who she knew of...’  ‘At blue cross, client refused to see either of my Asian colleagues and would only talk to me’ |
| Racism against Gypsies/Irish Travellers | 5 | ‘I [a student of Gypsy/Irish Traveller ethnicity] was called a **dirty smelly tinker** by a member of the public’    ‘Member of the travelling community came into the clinic and **all staff were paged to remain alert** and not to leave them unattended in the consult room or waiting room because they would rob something.’  ‘Being derogatory about someone's background (**a client who was a Gypsy**) to other members of staff’  ‘Racist remarks against travellers’  ‘At a farm animal practice, **I watched how vets/nurses reacted towards foreigners (ie immigrants/gypsies**).’ |
| Favouring Caucasian students over BAME students | 3 | ‘**Vets and vet nurses would ignore my questions and would only ever talk to me if they want me to do things for them** (ie doing laundry, feeding patients). **They are more likely to give learning opportunities to my caucasian classmates**, like offering them to do surgeries. This obviously impacts my learning. And one time during consult, a client asked if I was following what is happening, as **they assume I don't speak English.**’  ‘…[A] vet thinking he's being helpful (but actually came across pretty inappropriate and embarrassing for student involved) .. **pointed out the only foreign student in the room and stated that they are going to have a hard time being respected by clients as they look young and are foreign**, and stated it is their responsibility for the sake of the animals not to let this affect them...’  ‘Placement would not take student due to asian ancestry’ |

**Table 4 Common codes in students’ 'Other' reasons for not reporting discrimination**

| Code | Frequency | Examples |
| --- | --- | --- |
| Feeling the discrimination was not important enough to report | 9 | ‘…Figured there would always going to be someone in the work place that you won't click with and **sexism wasn't an important enough issue to report**.’  ‘…I also **didn’t think that it was severe enough** that it was worth reporting.’  ‘**I don’t report it because it’s not that big of a deal**. It’s just really really tiresome.’  ‘He was making a joke so **I felt that it would be an over reaction from my part if I reported it**’  ‘**I was not sure if it is actually discrimination against my race, and at times I feel like I've made those scenarios much worse in my head than they actually are**, and was a little worried that if I do report it, I would bear the consequences.’  ‘**Didn’t think it was such a massive deal** although the vet in question bullies other people in the practice’  ‘**Worried that people would tell me that I am just overthinking it**.’  ‘Generally they were quite subtle things/ comments, so **it didn’t feel substantial enough to report**. Also only at these places for 1 week, so just seemed easier to wait it out and move on.’  ‘**It did not feel important enough**; I did not feel like I needed action taken’ |
| Feeling unable to speak up due to position as an EMS student | 7 | ‘**I didn’t feel it was my place to report it as a student**. It was a client of a farm practice and as a guest to the practice it didn’t seem like my place’  ‘**Didn't feel able to raise it** with the staff of the practices **due to my position as a visiting EMS student**.’  ‘…**I wasn't secure enough in my environment on EMS to [report discrimination]**, but hopefully in the future I will be. However I don't think reporting this would have done very much seeing as **I felt too vulnerable to defend myself** at the time.’  ‘**Being a student on ems you feel like you have to fit into vets acceptable bracket of how a student should behave**. You don’t feel like you can say anything but awkwardly laugh.’  ‘I told my colleagues about this encounter but **I felt pretty powerless about the whole thing**. **I didn’t feel confident that I could report it directly to the senior vet** about the associate…’  ‘unsure if it was my place as a student’  ‘Felt I was not in a position to do so as a 3rd year EMS student there for one week.’ |
| Concerns about how they would be perceived if they reported | 5 | ‘**Didn't want to come off as soft** and unable to work with a wide range of people. ..’  ‘A general number one rule of veterinary practice is to keep the nurses on your side**. I doubt they would be on my side if I reported them** all for something like this..’  ‘**Didn't want to make a fuss in predominantly white environment**…’  ‘I generally accept that farm stuff to be a well known part of doing EMS, and **feel that reporting just gives them more belief women are more emotional than men**. ‘  ‘It’s a local vet and wouldn’t want to have to see him around after complaining. **I also wouldn’t want it to affect the other local vets perception of me should he talk about it with anyone**…’ |
| EMS requirements of the vet course coming before reporting | 4 | ‘**Because it is so crucial to undertake a vast amount of EMS I just feel like I should shut up and deal with it. No one cares**. I have had incidents in the past during EMS and friends have had them too and the response from the university was less than satisfactory, even going to blame them for what had happened despite the student being verbally and mentally abused for their sexual orientation. **I do not trust the systems in place to care. All they care about is the image of the university and if people complete their 26 weeks of EMS**.’  ‘… **my [EMS] form was still getting filled out** at the end of the day.’  ‘I felt I couldn’t say anything as it was the boss who was making the comments … **I figured if I spoke up I’d be asked to leave the placement or I would get a bad review**. I didn’t see how speaking up would have made a difference at the time to his behavior.’  **‘Don't want to take away EMS opportunity from a future student - fore warned is fore armed**.’ |

**Table 5 Responses to final question where students were invited to add any further thoughts/opinions – which show ambivalence/apathy/denial of diversity and discrimination as an issue in the veterinary profession**

| Student responses showing ambivalence/apathy/denial of diversity and discrimination as an issue in the veterinary profession |
| --- |
| ‘In terms of my answer to racial diversity; [referring to ‘strongly disagree’ response to Likert scale about need to increase racial diversity] I believe that those who are more suited for the course should apply, no matter what their racial/sexual/gender status is. **If we assign a minimum amount of racial diversity then you’re putting potentially better suited vets out of a career to satisfy a ridiculous quota.** As a boy, it always sits at the back of my mind If I actually would have gotten in this was a male dominated course.’ |
| ‘**It is very rare that I personally witness true discrimination**. True as in actually treating someone unjustly because you don't like how or when they were born (race, sex, orientation, age). I do see people getting offended a lot which is different. **Maybe you are offended by many statements or attitudes but they do not [fulfil] the definition of discrimination**. I think people tend to blur these two things into one and they should remain separate ideas.’ |
| ‘… **I also do not believe racial diversity needs to be increased in vet school as obtaining a place in vet school should be from merit** and I disagree that someone would have better chances to get in purely to improve diversity, similarly with gender (due to not as many boys at vet school). **I believe the only thing that should be looked on for improving someone chances is if they are from a poorer background**- not as good schools etc.’ |
| ‘The response you receive from other members in the practise is based on your enthusiasm, how you conduct yourself and how you treat others.’ |
| ‘In terms of the need to diversify, **I don't know how much that is a statistical need or [professional] need. I see few minorities in my peer group,** but **I also think most of us are more sensitive to the beliefs of different social group compared to the general population**.  In general, I think the student body puts in a lot of effort into supporting the LGBTQ+ community, and that the university has followed that lead.’ |
| ‘I think sometimes **vet students do just need to use their backbone a bit and suck it up more** especially when one day they will be graduated and they can choose which ever practice they want to work in.’ |
| ‘I have not witnessed a discrimination issue in the veterinary profession. I have not personally experienced any discrimination, nor have I witnessed any discrimination toward any of my friends/classmates all of whom are from diverse backgrounds and practice various ways of life and enjoy varied personal lives. **I believe the governing bodies are doing plenty to stop discriminating because it is under control and not an issue**. My opinion may differ from others because you did not establish what "discrimination" was. The **dictionary definition is: The act, practice, or an instance of discriminating categorically rather than individually**. Some may choose to broaden this and see more discrimination. **Under actual definition of the word I do not see a discrimination problem in the veterinary profession**.’ |
| ‘**Attitude and the interest shown by the student often earns them respect**, it's not necessarily that older students are more respected and not discriminated in clinics but more so their level of participation and knowledge.  unintentional discrimination based on level of skill or willingness to do certain tasks can also occurs in clinic, **perhaps not with mal intent but just a forgetfulness to include the student on placement.**’ |
| ‘I haven't experienced it myself and neither have my [closest] friends. I have heard stories retold by other people but never know how much truth there is in those.’ |

Table 6 Responses to final question where students were invited to add any further thoughts/opinions – which show consciousness of the importance/concern about diversity and discrimination in the veterinary profession

| Student responses showing consciousness of the importance/concern about diversity and discrimination in the veterinary profession |
| --- |
| ‘**We always talk about the gender unbalance in vet school, but NEVER the racial inequality - while the proportion of 'black, asian and minority ethnic' people in the England and Wales is 14% (2001), number of BAME people within the veterinary profession is 3%.** Huge skewed population within vet school, including an over-representation of middle and upper class students compared to lower and working class (race also intersects with this factor too as BAME people make up bigger proportion of the working class now).  So while it's important to keep pushing for women's equality, we should definitely not be neglecting pushing for racial, class and LGBT at the same time (if not more), because we're not the only ones who are held back.’ |
| ‘**Honestly, being at university has made me very aware of my gender, race and class. I think it has been cumulative from small comments here and there from other vet students or university students. Most discriminatory comments about vet students are from within the vet student community**.  **With some vet students there is a lack of exposure to certain cultures** which kind of perpetuates the problem as they don't know how to act around you (especially in 1st year). Some of them do hold certain perceptions of ethnic minorities in the UK and it is worrying.’ |
| ‘**I think with regards to the issue of the lack of racial diversity at vet school you'd to first address the lack of socioeconomic diversity**. The majority of people (at least in my year, at my uni) are from wealthy middle class backgrounds. **There are a lot of barriers to getting into vet school if you're from a working class family or lower income area**.’ |
| ‘I don’t feel like I can comment on the extent of racial/homosexual discrimination as a heterosexual white person’ |
| ‘I have not seen or known anyone who has seen/be [victim] of any vet student/vet discrimination personally and so don't know the extent of discrimination [particularly] racial or LGBTQ+ discrimination, however I understand that this is something that still affects the veterinary profession as well as most other professions’ |

Table 7 Respondents’ opinions regarding responsibility for/solutions to the issue of discrimination against veterinary students

| Students opinions regarding responsibility for/solutions to the issue of discrimination against veterinary students |
| --- |
| ‘…In general on my clinical EMS, I have found veterinary staff to be very respectful of other cultures. I'm half Asian and a farmer once interrupted me mid-sentence to ask if I could speak English (which I was already speaking at that exact moment...), **to which the vet immediately replied "better than you and me mate". Vets also protected me from going to certain farms if the farmer was notoriously sexist, racist or not very nice**, and the female vets and racially diverse vets were very good at sticking up for themselves against sexist/racist farmers and **would simply leave the farm immediately if they were discriminated against**.’ |
| ‘This is huge issue and one person reporting [can’t] make a difference. **It needs to be discussed on a professional level and find ways to encourage diversity in veterinary’** |
| ‘It would be nice to know what our outlets are for reporting discrimination’ |
| ‘**Honestly I don't think governing bodies can do much in tackling sexism in farm animal practice as the ideas of females being unable to cope is a generational idea that is engrained** in older farmers and is quite difficult to change. **It has to come from the practices themselves to generate this change**. **And not just one vet but all vets in the practice need to stand up for each other if they experience sexism/hear comments that are sexist**. **It is difficult for students to raise issues as they are being hosted by the practice**. You are only there for 2 weeks most of the time so often I think students will just ignore it rather than raise a concern because they know they will be leaving in a short time and probably won't have to see the sexist client/member of the public again.’ |
| ‘…**Wouldn’t know how to involve RCVS in discrimination incidents** **but would be comfortable talking to Glasgow uni** if it happens to me.’ |
| ‘**There should be more support for students on placements where they can turn to for advice in difficult/discriminatory instances**, or if they witness something illegal occurring at the practice.’ |
| ‘**I think it is very hard to ask governing bodies to mediate what members of the public say, what *all* staff say** in unusual or rural EMS, and what is said on EMS overseas. **I wish vet students got better information of how to handle the variety of discrimination we get but in a non judgmental way. Don't just tell me to report it and make me feel like I'm turning back feminism if I don't. Tell me some approaches of what I can do**, or pull together examples from others and what they do. I use humor a lot and clapback at them, but some types of passive aggressive or subtle discrimination are harder to point out (/report if you choose) but can still affect an EMS placement.’ |
| ‘The issue is more with the public than within the profession although **not enough is done to support/protect vet staff**’ |
| ‘**University's need to stop conditioning us to sit and take discrimination and bullying**. From first year we are taught to do what we are told **and not moan as it will look bad on us and the University**.’ |
| ‘I personally haven’t felt I’ve been discriminated against, but I will say that if I had been **I’m not sure I would know who to report it to. It’s not something that is discussed much at my school, and therefore I feel there is a lack of resources available**.’ |
| ‘While the vet school tries to be supportive of students, some find themselves in a bad situation. **When wanting to leave the situation, the vet school is quick to answer that students need to learn to work with people they dislike.** This forces the student to choose between a penalty or toxic working environment.’ |
| ‘**Unfortunately because there are such strict EMS weeks to fill I feel like vet students are scared to leave placements that make them feel uncomfortable for fear of missing out on required weeks** and having to make them up in another way. It would be helpful for EMS to be more structured. Having to find your own placements which **aren't vetted by the school or RCVS can lead to poor experiences**. The point of EMS is to gain experience and build relationships with professionals but I feel this rarely happens in the vast majority of placements.’ |
| ‘**I believe in the case of vet practices offering placements [to] students, there should be a "driving licence" of the type that students are required to do before being able to go to placements**. **Practices should have a minimum standard to which they are held to by the governing bodies such as the RCVS for the training of future vets**, including how they treat vet students and teach them in a way to build up rather than destroy confidence. If it is a requirement from universities that all students have experience in equine, smallies and farm, and by the RCVS that all students should have day 1 competencies in every field, vet practices should not [discriminate] based on what field they think the student should go into.’ |
| ‘Sadly, being a female gets you discriminated against on farms whether you are a vet student or qualified vet; it is very sad, and **I wish the profession could do more about it**.’ |
